# Contribution of natural antisense transcription to an endogenous siRNA signature in human cells

**DOI:** 10.1186/1471-2164-15-19

**Published:** 2014-01-13

**Authors:** Andreas Werner, Simon Cockell, Jane Falconer, Mark Carlile, Sammer Alnumeir, John Robinson

**Affiliations:** 1 RNA Biology Group, Institute of Cell and Molecular Biosciences, Newcastle University, Framlington Place, Newcastle NE2 4HH, UK; 2 Bioinformatics Support Unit, The Medical School, Newcastle University, Framlington Place, Newcastle NE2 4HH, UK; 3 School of Life Sciences, Northumbria University, Ellison Place, Newcastle upon Tyne NE18ST, UK; 4 Faculty of Applied Sciences, University of Sunderland, Wharncliffe Street, Sunderland SR1 3SD, UK; 5 Institute of Cellular Medicine, Newcastle University, Framlington Place, Newcastle NE2 4HH, UK

**Keywords:** Endo-siRNA, Noncoding RNA, Antisense transcripts, RNAseq

## Abstract

**Background:**

Eukaryotic cells express a complex layer of noncoding RNAs. An intriguing family of regulatory RNAs includes transcripts from the opposite strand of protein coding genes, so called natural antisense transcripts (NATs). Here, we test the hypothesis that antisense transcription triggers RNA interference and gives rise to endogenous short RNAs (endo-siRNAs).

**Results:**

We used cloned human embryonic kidney cells (HEK293) followed by short RNAseq to investigate the small genic RNA transcriptome. 378 genes gave rise to short RNA reads that mapped to exons of RefSeq genes. The length profile of short RNAs showed a broad peak of 20-24 nucleotides, indicative of endo-siRNAs. Collapsed reads mapped predominantly to the first and the last exon of genes (74%). RNAs reads were intersected with sequences occupied by RNAPII or bound to Argonaute (AGO1 by crosslinking, ligation, and sequencing of hybrids, CLASH). In the first exon, 94% of the reads correlated with RNAPII occupancy with an average density of 130 (relative units); this decreased to 65%/20 in middle exons and 54%/12 in the last exon. CLASH reads mapping to multi-exon genes showed little distribution bias with an average of about 5 CLASH reads overlapping with 60% of the endo-siRNA reads. However, endo-siRNAs (21-25 nt) intersecting with CLASH reads were enriched at the 5′end and decreased towards the 3′end.

We then investigated the 378 genes with particular focus on features indicative for short RNA production; however, found that endo-siRNA numbers did not correlate with gene structures that favor convergent transcription. In contrast, our gene set was found notably over-represented in the NATsDB sense/antisense group as compared to non-overlapping and non-bidirectional groups. Moreover, read counts showed no correlation with the steady-state levels of the related mRNAs and the pattern of endo-siRNAs proved reproducible after an induced mutagenic insult.

**Conclusions:**

Our results suggest that antisense transcripts contribute to low levels of endo-siRNAs in fully differentiated human cells. A characteristic endo-siRNA footprint is being produced at sites of RNAPII transcription which is also related to AGO1. This endo-siRNA signature represents an intriguing finding and its reproducibility suggests that the production of endo-siRNAs is a regulated process with potential homoeostatic impact.

## Background

The full nature of the human transcriptome is taking shape thanks to highly efficient sequencing strategies that reach unprecedented depth [1]. Layers of long and short RNAs with unknown biological functions keep emerging [2]. A particularly intriguing family of non-protein coding RNAs are natural antisense transcripts (NATs) [3]. NATs are commonly understood to be transcribed from the complementary DNA strand of protein coding genes. Processing of the primary antisense transcripts result in mRNAs that share complementary exons with the related sense transcript [4]. Genomic loci that express NATs are highly abundant and sense/antisense transcript pairs tend to be co-expressed [5,6]. The most comprehensive studies predict that in human and mice 40-72% of all transcriptional units show evidence of bi-directional transcription [7,8]. NATs are most prominently found in testis, they are also detectable at low levels in other tissues [5-7]. Interestingly, the occurrence of NATs correlates with genes that show imbalanced allelic expression (random imprinting or random monoallelic expression) [9]. This observation suggests that NATs carry the potential to induce allele-specific gene silencing. Accordingly, they are significantly under-represented on the mammalian X chromosome [8,10]. SiRNAs can potentially trigger transcriptional gene silencing and, interestingly, endo-siRNAs originating from sense/antisense RNA pairs have been detected in several model systems; however, the nature of these endo-siRNAs has not been thoroughly investigated [11,12].

The potential of NATs to form RNA-RNA hybrids with the sense transcript can trigger various mechanisms and regulatory cascades. The three best supported ones are RNA masking, the establishment of chromatin marks and RNA interference. RNA masking describes a process where the antisense transcript occludes a regulatory motif in the sense RNA by direct base pairing. Depending on the nature of these motifs the interactions stabilize or de-stabilize the mRNA [13]. Well-documented examples include the hypoxia induced factor 1α (HIF 1α) and β-secretase [14,15].

Chromatin modification as a result of ectopic expression of NATs has been linked to human disease [16,17]. A rare form of α thalassemia is caused by a genomic deletion that brings a constitutively active gene (*LUC7*) into close vicinity to the *HBA2* gene. The resulting antisense transcript was shown to induce methylation of a GC island in the promoter of *HBA2* and silence the gene [16]. In addition, a tumor suppressor gene (*p15*) was shown to be epigenetically silenced by antisense transcription. *P15* is repressed in a variety of cancers and an inverse expression of sense and antisense transcripts was discovered in leukemic cells [17].

The involvement of NAT-triggered RNA interference in gene regulation is supported by research focusing on the Slc34A gene (encoding a Na-phosphate cotransport protein). In this case, endo-siRNAs derived from sense/antisense overlaps were found in mouse testis and kidneys [9,18]. Moreover, large scale sequencing approaches also found endo-siRNAs originating from bi-directionally transcribed loci [11,12].

RNA interference involves two key enzymatic components, an endo-ribonuclease and an effector protein complex. The endonucleases, Dicer or Drosha, process double-stranded RNA precursors into short RNA duplexes of about 22 base pairs. These oligonucleotides are integrated into effector complexes that include an Argonaute protein [19,20]. One of the RNA strands is unwound and becomes quickly degraded [21]. The remaining single stranded RNA molecule, the guide strand, directs the RNA-protein complex to its biological target. RNA interference was initially thought to be a predominantly cytoplasmic process; however, recent studies have detected Dicer and Argonaute proteins in the nucleus associated with chromatin. Moreover, Argonaute-dependent processes have been documented to alter chromatin marks and also the dynamics of RNA polymerase II was shown to be affected by Argonaute and Dicer [22].

A putative link between antisense transcription and the synthesis of endo-siRNAs has not been comprehensively investigated. We used total RNA from cloned of HEK293 cells to determine the short RNA transcriptome. The analysis focused on reads that mapped to exons of protein-coding genes. Our findings suggest that sense/antisense transcripts can feed into an RNA interference related pathway. The resulting low levels of endo-siRNAs contribute to a stable short RNA signature in differentiated somatic cells.

## Results

### Analysis of endo-siRNAs

**Figure 1:**
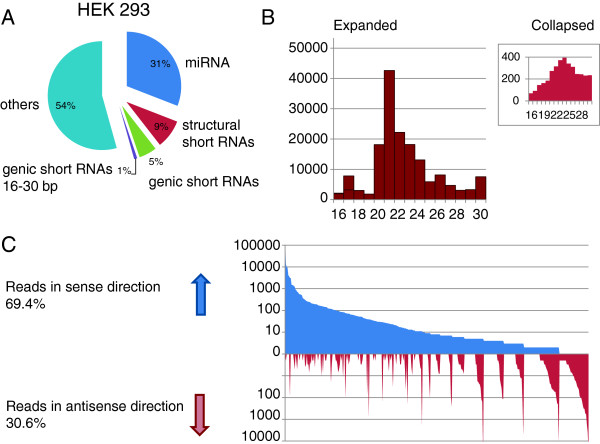
**Genic endo-siRNA in HEK293 cells. (A)** Pie diagram of the different families of short RNAs found in the RNA sample. **(B)** Length distribution of endo-siRNAs. The length of the RNAs is given on the x-axis, the read counts are on the y-axis. **(C)** Relationship between the orientation of the short RNAs and their genes of origin. The reads of each gene were grouped according to their orientation and then sorted according to the number of reads. The x-axis represents the short RNA reads sorted according the orientation of the reads. The y-axis represents the read counts (logarithmic scale).

To avoid cell heterogeneity due to the accumulation of stochastic mutations we first cloned HEK-293 cells by serial dilution. A single clone was selected and expanded for short RNA sequencing. Total RNA was isolated, size selected and then used to generate a directional cDNA library. The material was amplified and sequenced on an Illumina HiSeq 2000. A total of 30.9 million parsed reads were obtained and annotated to the human genome (hg18). The most prevalent of the genic short RNAs were microRNAs (31%) and short structural RNAs (9%). Genic short RNAs comprised 5% of the reads of which 1% (282319) were between 16 and 30 bases long (Figure 1A). In total, 378 genes gave rise to genic short RNAs (1.9% of all protein coding genes [Gencode version 18], Additional file 1: Table S1). The genes are localized on all but the Y chromosome which is not present in HEK293 cells. Chromosomes 5 and 11 contain a proportionally high number of genes with short RNAs (Additional file 2: Figure S1). Because of the relatively small size of the remaining data set the analysis was performed using the UCSC table browser and spread sheet functions. Size distribution of the short RNAs shows a broad peak between 20 and 24 nucleotides which concurs with the size of endo-siRNAs (Figure 1B) or 21-26 nucleotides if collapsed (Figure 1B, inset). The majority of endo-siRNAs reads were found to be in sense orientation with respect to the parent gene (69.4%), 30.6% were antisense (Figure 1C). Similar observations have been reported, indicating that preferentially the sense transcript gives rise to short RNAs [11,12]. Moreover, only 6.3% (24 of 378 genes) displayed significant read counts (>5) in both sense and antisense orientation. Of note, manual scrutiny of the 378 genes revealed that 3 genes, TMEM25, LPPR5 and LYPD3, contributed disproportionally to the dataset with almost 63% of the reads. These 3 genes have exonic hairpin structures that feed into micro RNA processing (hsa-miR151A, LPPR5 and LYPD) or are possibly related to piRNAs (TMEM25) and are therefore excluded from the quantitative analysis of expanded endo-siRNA reads.

First, we investigated putative sources of genic endo-siRNAs; stalled RNA polymerase II (RNAPII) and the RNA interference pathway both being linked to the genesis of these RNA species. To assess the involvement of RNAPII we downloaded the publicly available RNAPII ChIP-Seq data (chromatin imunoprecipitation-sequencing) from the GEO database (http://www.ncbi.nlm.nih.gov/geo/query/acc.cgi?acc=GSM891237) [23].

**Figure 2:**
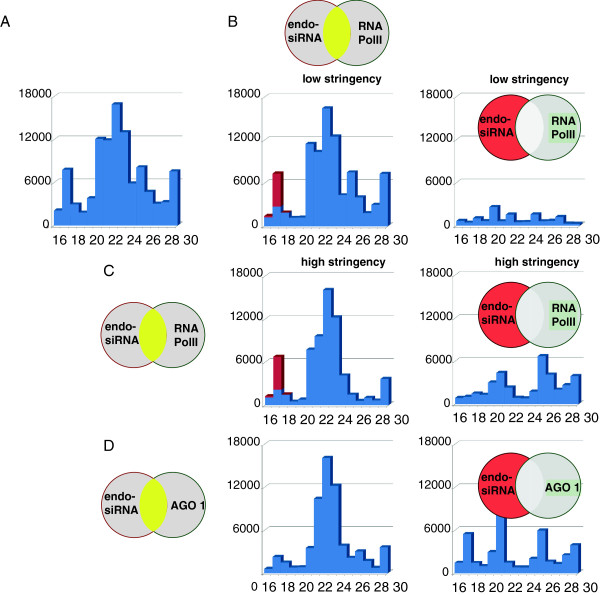
**Length signature of endo-siRNAs that co-localize with RNAPII or co-precipitate with AGO 1. (A)** Total endo-siRNA sample excluding reads that map to LPPR5, LYPD3 or TMEM25, as discussed in the text. **(B)** Endo-siRNA signature of all reads that do (left) or do not (right) co-localize with RNAPII as indicated by the Venn diagram. **(C)** Same as **(B)** but reads that mapped to regions with low RNAPII ocupancy (<1.5 percentile) were excluded, low stringency. **(D)** Endo-siRNA length distribution of reads that do (left) or do not (right) associate with AGO1. The rust-colored reads stem from a single gene, STAMBP.

Collapsed reads were manually binned into 4 categories, first 5′ exon (25.3%), middle exons (22.5%), last 3′ exon (37.8%) and single exon genes (10.6%) using IGV (Integrated Genomics Viewer [24,25]) followed by intersection with RNAPII occupancy using the UCSC table browser. As expected, both co-localization (94.3% of the reads covered sequences with RNAPII occupancy) as well as signal strength (129.6 average integrated units) was highest for reads mapping to the first 5′exon. These values decreased to 65%/20.4 integrated units in internal exons and 54.4%/12.1 units in the final exons. Single exon genes showed average occupancy and intensity (65.7%/54.2) (Table 1, upper panel). Next, the reads mapping to RNAPII occupancy were quantified with respect to their length; profiles were established for all reads intersecting with RNAPII occupancy (Figure 2B, low stringency) as well as for reads that map to highly RNAPII occupied regions (excluding the lowest percentile, <0.72 units) (Figure 2C, high stringency). Short RNAs of 21-25 nt were found to predominantly map with RNAPII occupied sites in addition to lesser populated length bins (Figure 2B, left panel). Interestingly, increased stringency predominantly depleted RNAs of 20-22 nucleotides and of 26-30 nucleotides from the data set (Figure 2C right panel). The peak of 21-25 nt was notably sharper (Figure 2C, left panel) indicating a possible weak association between components of the RNAi machinery and RNAPII [26].

**Table 1:** Summary of intersections between endo-siRNAs and RNAPII occupied sequences (top) and AGO1 CLASH data (bottom)

**Intersection RNAPII and short RNA reads**					
	**Integrated units**	**Total siRNA reads**	**Average**	**Reads with RNAPII occupancy**	**%**
Total reads	250197.3	4628	54.1	3236	69.9
5'	146555.5	1131	129.6	1067	94.3
3'	20395.1	1687	12.1	917	54.4
Internal exons	20449.3	1003	20.4	652	65.0
1 exon genes	25726.7	475	54.2	312*	65.7
Others	3106.0	332	9.4	122*	36.7
**Intersection AGO1 CLASH and short RNA reads**					
	**CLASH reads**	**Intersected siRNA reads**	**CLASH/siRNA** **reads**	**Total siRNA reads**	**%**
Total reads	22073	2572	8.6	4628	55.6
5'	3143	686	4.6	1131	60.7
3'	5621	1037	5.4	1687	61.5
Internal exons	2707	562	4.8	1003	56.0
1 exon genes	9070	117	77.5	133*	88.0
Others	1385	133	10.4	162*	82.1

The star (*) indicates discrepancies in “total siRNA reads” between top and bottom panel that arose from conversion of the CLASH data from Genome assemblies hg19 to hg18.

**Figure 3:**
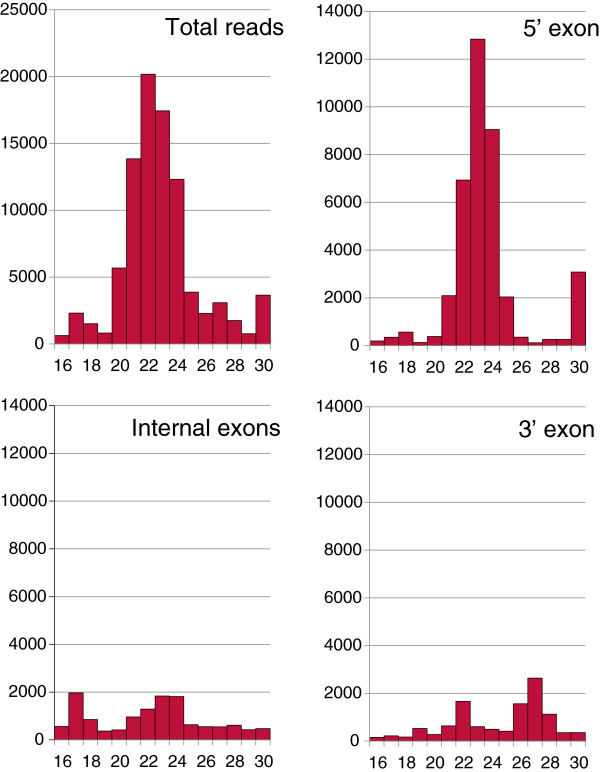
**Length signature of endo-siRNAs intersecting with AGO1 CLASH reads.** Diagrams show the total reads (upper left) binned according to their length (16-30 nucleotides), reads in the first exon (upper right), in internal exons (lower left) and in the last exon (lower right) of genes.

Next, the collapsed and binned reads were intersected with a CLASH (UV cross-linking and analysis of cDNAs) dataset that was generated using an antibody against AGO1 and UV treated HEK cells, kindly provided by A. Helwak and D. Tollervey [27,28]. We found that about 60% of the endo-siRNA reads overlapped with an average of about 5 (expanded) CLASH reads throughout multi-exon genes (Table 1, lower panel). Single exon genes, constituting predominantly histone genes in this dataset, showed a markedly different coverage with an 88.0% overlap between the two datasets and 77.5 CLASH reads per collapsed endo-siRNA. There was again evidence for an interaction between the RNAPII machinery and AGO1 as the endo-siRNA peak of 21-25 nucleotides gradually decreased from 5′ end to internal exons and 3′end (Figure 3).

### Convergent transcription

In order to test the hypothesized link between convergent transcription and endo-siRNAs we compiled characteristic parameters of each individual gene associated with endo-siRNAs. The argument was that convergent transcription of genes in close vicinity will favor endo-siRNA production. First, the distance to both neighboring genes was determined by subtracting the end coordinate of the last exon from the start coordinate of the first exon of the following gene. The parameters collected of the 378 genes included orientation, length and exon-number, distance to neighboring genes and their orientation as well as the number and orientation of reads. The finding, that 30.6% of the genic reads were oriented in antisense to the transcript of origin may suggest a role for antisense transcription in generating endo-siRNAs.

**Figure 4:**
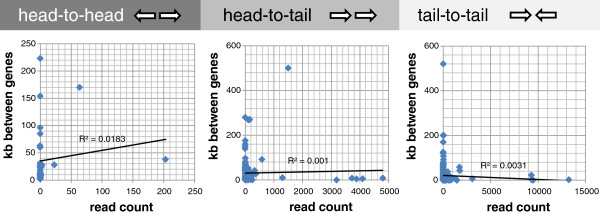
**Scatter plot of endo-siRNA read count versus distance to the neighboring gene.** Left panel, genes arranged head to head (divergent transcription); middle, genes in tail to head orientation; right panel, genes in tail to tail arrangement (convergent transcription). The R^2^ values indicate a lack of correlation in all three panels.

We sorted the 176 genes with a near neighbor in tail-to-tail configuration according to the distance between the two genes to assess if the potential antisense transcript promotes the formation of endo-siRNAs. As Figure 4 demonstrates, there is no link between distance and configuration of neighboring genes (head to head, tail to head and tail to tail) and the number of endo-siRNA reads. The low R^2^ values suggest that there is no direct correlation between the convergent transcription of closely located genes and endo-siRNAs. Other parameters such as the length of the endo-siRNA producing genes and their exon count had no influence on the number of endo-siRNA reads (not shown).

To obtain a better estimate concerning the role of antisense transcripts in establishing the endo siRNA signature we tested the classification of the 378 loci according to the antisense database NATsDB ([29]; http://natsdb.cbi.pku.edu.cn/). We found the sense/antisense (SA) category over-represented in our dataset as compared to the overall distribution (53% versus 42.2%). The non-bidirectional gene category was reduced (41.7% versus 52.9%) whereas the bi-directionally transcribed but non-overlapping loci were represented equally in both datasets (5.3% versus 4.9%; Table 2).

**Table 2:** Representation of endo-siRNA related genes in the NATsDB antisense database

	**Endo-siRNA linked genes**	**%**	**Total gene names**	**%**
Non Bi-Directional (NBD)	133	41.7	12733	52.9
Non Overlapping SA (NOB)	17	5.3	1170	4.9
Sense/Antisense (SA)	169	53.0	10150	42.2

Gene names and accession numbers were used as identifiers, however, a small number of genes (59) were not found in none of the three categories NBD, NOB and SA. The right panel gives the proportion of single categories for all genes.

An intriguing observation was made related to genes in tail-to-tail orientation on the X chromosome. Either such gene pairs were separated by substantial gaps (> 35 kb) or they were located at chromosome ends in clusters with relaxed imprinting. This finding concurs with the documented reduced expression of antisense transcripts from the X chromosome and suggests that in a biologically relevant cellular context convergent transcription may be linked to epigenetic gene silencing.

**Figure 5:**
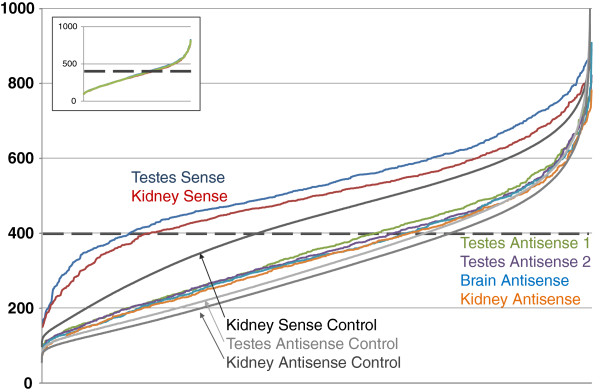
**Expression level of endo-siRNA related genes in sense and antisense direction in different tissues.** Expression data is from Ling et al. [5], probe sets that intersect with the endo-siRNA reads are shown. Probes are ranked according to their intensities, values >400 are considered as significant expression [5]. Expression of sense transcripts in testes (blue) and kidney (red); expression of antisense transcripts in testes repeat 1 (light green), testes repeat 2 (light violet), brain (light blue) and kidney (yellow). The entire transcriptomes of kidney sense (black), testes antisense (light grey) and kidney antisense (dark grey) were used as controls. The inset shows the curves of the average testes sample from two replicates (green), mean plus standard deviation (blue) and mean minus standard deviation (red). A representation with a fixed order of the kidney expression data and tissue dependent variations of the same gene in testes and brain results in a comparable but noisier outcome (Additional file 3: Figure S3).

We also assessed the expression levels of endo-siRNA related genes using the natural antisense database (http://bioinformatics.sdstate.edu/datasets/2012-NAT/) [5]. Since this repository does not contain information on HEK293 cells, we used human kidney, brain and testes as references, instead. We tested the expression levels of probes that overlapped with endo-siRNA reads. As reported elsewhere [6], we found that sense transcripts were generally expressed at higher levels than antisense transcripts. Interestingly, the endo-siRNA producing transcripts are generally higher expressed than average, in both sense and antisense orientation (Figure 5, Additional file 3: Figure S3). These data have to be considered with caution because HEK cells may display variations in gene expression that are not reflected in renal tissue. On the other hand, the small inter-tissue variation suggests that the increased levels of expression are real –as one would expect based on the above established connection between endo-siRNA and RNAPII.

So far, we identified and characterized short RNAs in differentiated HEK293 cells which map to exons of protein coding genes. A significant number of endo-siRNAs correlate with RNAPII occupancy and AGO1 CLASH signals. Because SA loci are enriched in our dataset it is conceivable that convergent transcription contributes to the synthesis of these endo-siRNAs.

### Effect of mutagenesis on endo-siRNAs

In HEK cells more than 12’000 genes are expressed [30], only 378 of which produce endo-siRNAs. We wanted to investigate whether this endo-siRNA signature may be characteristic for a specific cell stage or whether it is an unstable, transient feature. To address this question we subjected the original HEK293 clone to random mutagenesis by ethyl methyl sulfonate (EMS) and immediately re-cloned the cells. Two individual mutagenized clones were selected and used for RNAseq (denoted C5 and C12). A total of 44.3 and 47.1 million parsed reads was obtained from C5 and C12, respectively; the data from the mother clone was included into the further analysis as a control. The reads were annotated to the human genome (hg18) and again categorized into “non genic” and “genic”, the latter group including miRNAs, structural RNAs (largely snoRNAs) and exonic reads. In general, both mutagenized clones followed very similar trends regarding up –or down- regulation of specific RNAs –in agreement with single allele point mutations induced by EMS. An overview of the results for all three clones (control, C5 and C12) is given in Table 3. The impact of EMS mutagenesis on a genome wide level was established by assessing the variance of read numbers mapping to the individual genes. The pooled variance was taken as readout for the systemic consequences of the mutagenic insult. RNAs involved in a tightly controlled network were expected show a small variance (miRNAs) whereas a highly redundant system would tolerate a large variance (snoRNAs, [31]).

**Table 3:** Summary of the RNAseq results

	**Wild type**		**Clone 5**		**Clone 12**	
		**% of total**		**% of total**		**% of total**
Total reads	30903553		44325908		47144485	
Total genic reads	14112356	45.67	26539818	59.87	35543718	75.39
**miRNA**	**9549312**	30.90	**21615459**	48.76	**29684876**	62.97
**Small structural RNAs**	**2646057**	8.56	**2252302**	5.08	**1984388**	4.21
Exonic reads	1916987	6.20	2672057	6.03	3874454	8.22
**Exonic reads 16-30 bases**	**282319**	0.91	**586555**	1.32	**627123**	1.33
Others	16791197	54.33	17786090	40.13	11600767	24.61

The categories in bold are further investigated in this project.

**Figure 6:**
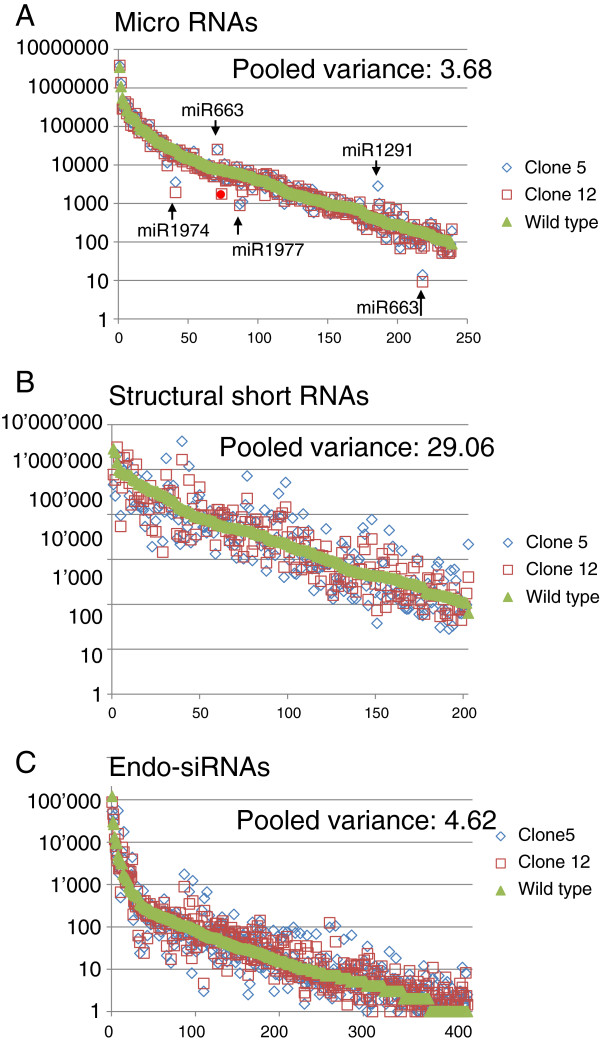
**Normalized read numbers of genic short RNAs in the three samples after EMS mutagenesis: wild type, clone 5 and clone 12.** The three panels represent microRNAs **(A)**, short structural RNAs **(B)** and endo-siRNAs **(C)**. In all panels, the wt sample is displayed in green, clone 5 is in blue and clone 12 in red. The data are sorted according to decreasing read counts along the x-axis, the y-axis represents the read counts in logarithmic scale. MicroRNAs with significant up -or down regulation are indicated in the graph **(A)**. Mir-1201, labeled with a red spot, overlaps with the annotated snoRNA (SNORD126).

The most prevalent of the genic short RNAs in both control and mutagenized samples were microRNAs, which is in agreement with published short RNAseq data. Hsa-mirs 10a/b, 182 and 92a-1/92a-2 made up more than 60% of all miRNA reads. In total, 238 different miRNAs scored higher than 100 reads in the control HEK293 cells and these were further examined. The miRNA expression pattern proved resistant towards mutagenic insults showing a pooled variance of 3.68 (expression change compared to wild type) (Figure 6A, Additional file 4: Table S2). In both mutagenized clones 86.5 and 87% of the genes showed less than 2-fold expression changes.

A total of 2.73 (control), 2.26 (C5) and 1.99 (C12) million reads mapped to 261 different structural RNAs, predominantly snoRNAs. In contrast to the stable miRNA transcriptome, the reads mapping to small structural RNAs showed considerable pooled variance of 29.06 in the mutagenized samples. Interestingly, the vast majority of the structural genes were down regulated (63.6 and 67.05%), only 27.59 and 30.27% remained stably expressed (Figure 6B, Additional file 4: Table S2).

The endo-siRNAs from the mutated samples were mapped and the pattern was compared to the control sample (Table 3). 586555 (C5) and 627123 (C12) reads of 16-30 bases were identified and shown to have comparable length distribution as the wild type (Additional file 5: Figure S2). Strikingly, we found a near perfect match of genes with annotated reads in all three samples. The pooled variance of all endo-siRNA reads in C5 and C12 was 4.62 (Figure 6C). In the two mutated clones comparable numbers of genes were significantly affected (22.8% and 5.77% up regulated, 15.11% and 16.21% down regulated; 62.09% and 78.02% remained unchanged).

**Figure 7:**
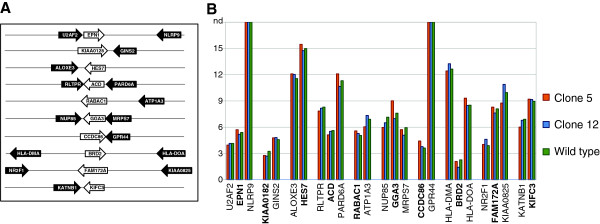
**Expression analysis of genes with short RNAs and their close neighbors. (A)** Schematic representation of the investigated loci. **(B)** RT-qPCR analysis of mRNA levels in wt, clone 5 and clone 12. The values represent the ΔcT values as compared to β-actin. The genes producing the short RNAs are in bold. The assays for the genes not detectable in HEK293 cells (NLRP9 and GPR44) were tested positive using CaCo-2 cells which express these genes. All the standard deviations are below 1 PCR cycle.

To test whether the changes in short RNA abundance influenced mRNA levels of the relevant genes we assessed the stable output of 10 loci by RT-qPCR. We selected loci that displayed clear changes in short RNA read numbers in both sense and antisense orientation and also quantified the output from the neighboring genes. The results are presented in Figure 7 and Additional file 6: Table S3. Most of these genes were found to be expressed at a significant level (thus supporting the findings in Figure 5) but the occurrence of endo-siRNAs was not related to their steady state expression level. We also tested the half-life of 5 selected mRNAs (FAM172A, EPN, ACD, TMEM25 and ACTB) and found no differences between the three clones (not shown).

To conclude, we have identified a layer of genic endo-siRNAs related to AGO1 and RNAPII occupancy. Experimental evidence suggests that convergent sense-antisense transcription contributes to the synthesis of these endo-siRNAs. The endo-siRNA signature is largely unaffected by mutagenic perturbation and does not reflect the level of the parent mRNAs. Because only about 2% of all expressed genes in HEK cells contribute to the RNA signature these endo-siRNAs are therefore unlikely to be a direct byproduct of transcription. Our observations suggest endo-siRNAs to have a biological role and add significance to recent RNA sequencing projects which report increasingly complex layers of low abundance short RNAs.

## Discussion

We present a comprehensive characterization of genic short RNAs in the human kidney cell line HEK293. We found that only a limited number of genes give rise to short RNAs, predominantly endo-siRNAs that are associated with AGO1 and correlate with RNAPII occupancy at 5′ends of genes. This RNA signature does not scale with the transcription of the related gene and expression level of the mRNA. Moreover, the short RNA pattern is resistant to mutagenic insults indicating that the endo-siRNAs are the result of a controlled synthesis –or the byproduct of a controlled process.

Three possibilities how these endo-siRNAs are being produced are plausible and not mutually exclusive. First, RNAPII produces a variety of RNA by-products, related to pausing of the enzyme during initiation and termination of transcription or as a consequence of incomplete splicing [32]. The most prominent of the short RNA species are denoted “promoter-associated small RNAs” (PASRs) and “transcription initiation RNAs (tiRNAs) [33]. Their occurrence is linked to highly active promoters and does not appear to involve RNA interference since they are either capped (PASRs) or do not co-precipitate with Argonaute (tiRNAs). tiRNAs are hypothesized to be generated by the backtracking RNA polymerase and the action of an intrinsic endonuclease activity [34]. The fact that the short RNAs identified in this screen show a very restricted expression pattern and do not scale with RNAPII occupancy genome-wide suggests that the RNA signature is not an obligatory by-product of transcription.

Second, the reads could represent degradation products from cellular mRNAs and reflect physiological mRNA turnover. Several observations, however, argue against this hypothesis. Only about 1-2% of genes produce short RNAs whereas roughly 40-50% of mRNAs generate positive calls in expression analysis. Moreover, careful expression analysis of 10 gene clusters, genes producing short RNAs and its neighbors (discussed below), showed no correlation between mRNA levels and short RNA reads. On the other hand, we identified genes where a very low number of randomly distributed reads could well be explained by RNA breakdown or sequencing errors, these were not studied in further detail. The reproducibility of the endo-siRNA signature after mutagenesis, however, suggests that such random errors do not contribute significantly to the sequencing output.

Third, the short RNAs identified in this screen could be synthesized by components of the RNAi machinery. Nuclear localization of key components such as Dicer, Drosha and Argonaute proteins and shuttling of related RNA protein complexes between the cytoplasm and the nucleus has been reported [35,36]. Our results from the AGO1 CLASH experiments and also the length profile of the short RNAs suggest that the RNAseq reads are significantly constituted of endo-siRNAs. At what stage a double stranded RNA precursor is formed and dicing occurs, is yet unknown.

One particular focus of the presented work was to investigate a potential link between convergent transcription, i.e. the co-expression of sense and antisense transcripts, and the formation of endo-siRNAs. Genic endo-siRNAs have been documented in several systems, predominantly in *C. elegans*, *Drosophila* and also in mouse [18,37-40]. However, in mammalian systems, the link between convergent transcription and RNA interference is controversial [41]. Our results confirm the existence of endo-siRNAs in human cells and the fact that 53% of these derive from SA loci provides circumstantial evidence that sense/antisense transcription may be involved. Our observation, that genes on the X chromosome tend to avoid the possibility of convergent transcription, indicates that situations of convergent transcription may indeed exist in somatic, diploid cells.

There is a substantial body of evidence that siRNAs can promote both transcriptional silencing and activation through chromatin modifications [42,43]. The siRNAs used in those experiments were synthesized *in vitro* and applied at high concentrations indicating that the process was inefficient. Our findings also suggests that the cell culture model used may not express balanced levels of all the components essential for the processing of NATs into endo-siRNAs and transcriptional silencing. For example, Hela cells transfected with vectors expressing both thymidylate synthase (TS) sense and antisense transcripts failed to generate TS related siRNAs [41]. On the other hand, highly expressed convergent transcripts from a single plasmid were recently demonstrated to produce siRNAs which induced transcriptional gene silencing in *trans*[44]. Our findings support the conclusion that the production of endo-siRNAs from NATs is inefficient in HEK293 cells and probably in other cell culture models as well. Indeed, the genome wide studies suggest that NATs-linked endo-siRNA processing is a highly cell-specific process, for example in developing sperm cells where antisense transcripts and endo-siRNAs are prominently found [6,45].

## Conclusions

In HEK293 cells convergent transcription may trigger the production of endo-siRNAs; however, the process appears to be rather inefficient and only relevant in specialized cell types. Moreover, in depth analysis of genic short RNAs revealed two interesting and novel features of the transcriptome: Firstly, we identified a distinct endo-siRNA signature that maps to a restricted number of genes and remains largely stable after a mutagenic insult. Secondly, read numbers of endo-sRNAs do not reflect steady-state mRNA levels of their parent genes.

## Methods

Cell culture, cloning and mutagenesis: The human embryonic kidney cell line HEK293 was maintained and passaged according to established cell culture conditions. Cloning was performed in a 96 well cell culture dish by serial dilution starting with 60-120,000 cells. Cells were grown until single colonies could be identified. Individual clones were transferred to 24 well plates and grown to confluency. At this stage a single clone was chosen and passaged into two wells of a 6 well plate. When the cells were about 80% confluent, the medium in one well was replaced and 100 μg/ml ethyl methanesulfonate (EMS; Sigma) was added. Cells were grown in the presence of EMS for 24 hours followed by a 24 hour recovery period in fresh medium. At that point the mutagenized cells were re-cloned by serial dilution. Thereafter, single mutagenized clones were expanded and two clones, C5 and C12 were randomly selected for further experiments. The non-mutagenized original clone was propagated to yield enough cells for RNA extraction and long term storage.

Nucleic acid isolation: RNA and DNA were isolated using Trizol according to standard methodology.

Short RNAseq: Short RNA purification was performed by GATC Biotech in Konstanz, Germany. In brief, the RNA was quality tested and size selected on a denaturing polyacrylamide gel (approximately 19-29 bases). Tagged 3′ adaptors and 5′ adaptors were ligated to the recovered RNA to ensure strand specificity. The material was used for cDNA synthesis. The three samples were pooled and sequenced on an Illumina HiSeq 2000. The sequencing data has been submitted to the European Nucleotide Archive, accession number GSE52996.

Data analysis: The reads were processed using the fastx toolkit to remove low quality reads and trim low quality bases. The parsed reads were mapped to the hg18 build of the human genome using Bowtie [46]. Samtools was then used to remove unaligned reads [47]. Refseq exons and micro RNA tracks were downloaded from the UCSC Genome Browser server and intersectBed from bedtools was applied to compare the reference data with the experimental data sets [48]. Overlaps were sorted into miRNAs, short structural RNAs and genic RNAs. Reads of more than 30 bases or less than 16 bases were removed from the genic RNA data set. Further analysis was done with standard spread sheet programs using specific statistical functions to collapse reads, sort reads according to their length and generate the graphs.

Reverse transcription- quantitative PCR: RNA from the original extraction, stored at -80°C, was used for expression analysis by RT-qPCR. Reverse transcription of approximately 0.5 μg of total RNA was performed using the Omniscript kit from Qiagen following the supplier’s instructions. In brief, RNA and 2.5 μM random hexamers were denatured for 3 minutes at 70°C and cooled to 37°C. The reaction mix including polymerase, dNTPs, buffer and RNase inhibitor were added. After one hour the reaction was denatured for 2 minutes at 95°C and stored at -20°C. 0.5 μl of the RT product was amplified in 1x Lightcycler 480 Probes Master mix (Roche) and gene specific PrimeTime® qPCR primers and probes (Integrated DNA Technologies). The cycling protocol included a denaturation step (95°C for 10 minutes) and 45 cycles of 95°C for 5 seconds, 55°C for 20 seconds and 72°C for 1 second when fluorescence was determined. The RT was performed twice with each RNA and the qPCRs were repeated and run in duplicates. qPCR reactions that resulted in a Ct difference of >1 between duplicates were repeated. The sequence of all the primers and the details about the PrimeTime® Assays are provided in Additional file 7: Table S4.

## Competing interests

The authors declare that they have no competing interests.

## Authors’ contributions

AW planned the study and wrote the manuscript. SC analyzed and mapped the RNAseq data, MC compared the data to deposited data sets. JF cloned and mutagenized the cells, SA performed the expression studies. JHR conceived of the study, and participated in its design and coordination. All authors read and approved the final manuscript.

## Supplementary Material

Additional file 1: Table S1 Compilation of genes producing short RNAs. Click here for file

Additional file 2: Figure S1 Genes with short reads compared to the total number of genes. Click here for file

Additional file 3: Figure S2 Expression of endo-siRNA related genes. Click here for file

Additional file 4: Table S2 Expression data of miRNAs and structural RNAs. Click here for file

Additional file 5: Figure S3 Length distribution of the short RNAs in the three samples control, clone 5 and clone 12. Click here for file

Additional file 6: Table S3 Summary of qPCR results. Click here for file

Additional file 7: Table S4 Details and sequences of primers and probes for qPCR. Click here for file
