# Supplementary material for: Contribution of natural antisense transcription to an endogenous siRNA signature in human cells
Source: BMC Genomics. 2014 Jan 13;15:19. doi: 10.1186/1471-2164-15-19 (PMC3898206; doi:10.1186/1471-2164-15-19)
Supplement: Additional file 1: Table S1 — Compilation of genes producing short RNAs. [file 1471-2164-15-19-S1.pdf]

Chromosome	Gene name	Gene length I	Number of exons	Orientation	Closest neighbor	Gene arrangement	Distance	Sense reads	AS reads
1	CNST	20	41	+	SCCPDH	T-H	55	1	0
1	INADL	420	39	+	L1TD1	T-H	31	5	0
1	NR_040077	370	16	+	WLS	T-T	0	5	0
1	HIST3H2BB	0.45	1	+				9	0
1	SESN2	23	10	+	MED18	T-H	46	12	0
1	HIST2H2AA4	0.6	1	-				18	0
1	MTR	106	33	+	RYR2	T-H	30	23	0
1	SKI	81	7	+	MORN1	T-T	11	27	0
1	IPO9	55	24	+	SHISA4	T-H	4.4	31	0
1	HIST2H3C	0.5	1	+				60	0
1	OTUD3	30	8	+	PLA2G2E	T-T	7.3	63	0
1	HIST2H2AA3	0.6	1	+				154	0
1	C1orf43	136	7	-	C1orf189	T-H	0.4	1	1
1	MSTO1	4.8	14	+	YY1AP1	T-T	49	1	1
1	F11R	26	10	-	ITLN2	T-H	40	1	2
1	ACOT7	130	12	-	GPR153	T-H	3.2	1	8
1	NCSTN	55	33	-	COPA	H-H	0	11	1
1	SPEN	94	15	+	ZBTB17	T-T	1.4	84	1
1	SERINC2	25	12	+	LOC149086	T-H	63	95	1
1	CNN3	27	7	-	SLC44A3	T-T	1.7	221	2
1	COPA	55	33	-	PEX19	T-H	3.4	3	0
1	FCRLB	5.5	6	+	DUSP12	T-H	23	1	3
1	PHC2	52	15	+	ZNF362	T-T	23	4	0
1	SLC1A7	56	11	-	PODN	T-T	1.7	4	0
1	OBSCN	170	61	+	TRIM11	T-T	15	2	4
1	SETDB1	39	22	+	CERS2	T-T	0.4	10	4
1	MAN1C1	166	12	+	SEPN1	T-H	15	1	5
1	HIST2H3C	0.6	1	-				6	2
1	FUBP1	31	20	-	NEXN	T-T	4	11	0
1	HIST2H2BE	2.2	1	-				23	1
1	EDARADD	90	7	+	LGALS8	T-H	32	4	24
1	PTCH2	23	23	-	BTBD19	T-T	5.5	27	1
1	NVL	103	22	-	DEGS1	T-T	34	28	142
1	ENO1	18	12	-	ENO1-AS	H-H	na	38	0
1	HIST2H4A	0.6	1	-				53	0

1	C1orf135	25	3	-	FAM54B	T-T	1	79	0
1	LPPR5	115	6	-	SNX7	T-T	125	0	30320
1	RHOU	101	5	+	RAB4A	T-H	500	0	146
1	IL12RB2	90	15	+	SERBP1	T-T	10.8	1	101
1	TOR3A	14	6	+	ABL2	T-T	3.4	38	696
1	GLUL	10	9	-				252	2
1	B4GALT3	7	8	-	PPOX	T-T	0.1	117	18
1	HIST2H4A	0.43	1	+				118	
2	AFTPH	69	10	+	LOC339807	T-H	14	1	0
2	LCLAT1	195	6	+	CAPN13	T-T	80	3	0
2	RPL31	17	8	+	TBC1D8	T-T	0	3	0
2	TRAF3IP1	80	17	+	ASB1	T-H	26	3	0
2	ATP5G3	5.6	3	-	ATF2	T-H	8	1	0
2	RASGRP3	126	20	+	FAM98A	T-T	18	1	1
2	RBM43	136	4	-				1	49
2	OSBPL6	200	23	+	TTN	T-T	0	0	2
2	WBP1	2.6	4	+	MGS	T-T	0.2	4	2
2	CCNYL1 (mir4775)	44	10	+	FZD5	T-T	6.5	248	2
2	NFU1	40	8	-	GFPT1	T-H	9	3	0
2	CEBPZ	28	15	-	NR_037879	T-T	0	4	0
2	DLX2	3.3	3	-	DLX1	T-T	9.5	7	4
2	C2orf72	12	3	+	PSMD1	T-H	7	1	22
2	FARP2	140	25	+	STK25	T-T	0.2	1	41
2	GLI2	194	13	+	TFCP2L1	T-T	200	1	54
2	USP34	286	55	-	AHSA2	T-T	0	191	0
2	EIF2AK3	71	17	-	C2orf51	T-T	27	1501	0
2	CUL3	115	16	-	FAM124B	T-H	68	195	0
2	CDC42EP3	28	2	-	QPCT	T-T	170	362	1
2	STAMBP	38	10	+	ACGT2	T-H	26	2	4797
3	C3orf78	4	2	+	PBRM1	T-T	4.6	2	0
3	RPP14	43	24	+	PXK	T-H	12	7	0
3	SMC4	35	25	+	TRIM59	T-T	0.5	10	0
3	CTNNB1	41	15	+	ULK4	T-T	6	16	0
3	EIF4A2	6.6	13	+	RFC4	T-T	0	21	0
3	CHCHD4	12	4	-	TPRXL / TMEM43	T-T /H-H	43 / 0.1	2	0
3	DHX30	47	23	+	MAP4	T-T	0.5	16	2

3	EIF2B5	10	16	+	DVL3	T-H	9.5	12	3
3	COMMD2	14	5	-	WWTR1	T-H	35	3	23
3	TOPBP1	61	28	-	CDV3	T-T	10	5	0
3	RNF123	28	36	+	AMIGO3	T-T	0	5	5
3	GMPPB	28	36	+	RNF123	T-T	0	4	18
3	DLG1	257	26	-	MFI2	T-H	13	10	0
3	TATDN2	33	8	+	GHRLOS2	T-H	3.2	8	15
3	LAMB2	12	32	-	USP19	T-H	0.2	28	0
3	RPL32	7	4	-	CAND2	T-T	0.1	3725	0
3	FAM55C	48	9	+	NFKBIZ	T-H	0	8	98
4	RBM47	205	7	-	CHRNA9	T-T	68	0	1
4	SLC4A4	387	14	+	GC	T-T	170	1	0
4	RPL34	10	6	+	OSTC	T-H	20	3	0
4	HPSE	42	13	-	COQ2	T-H	7.7	2	0
4	WHSC1	108	23	+	WHSC2	T-T	0.5	3	0
4	GRSF1	24	10	-	RUFY3	T-T	7.2	4	0
5	KIF2A	80	20	+	DIMT1	T-T	1.3	3	0
5	MARCH6	80	25	+	ROPN1L	T-H	6.5	4	0
5	FBXO4	16	7	+	C5orf51	H-T	3.5	4	0
5	SLC12A2	105	26	+	FBN2	T-T	68	4	0
5	SMA4	1200	15	-	RAD17	T-T	0	0	7
5	TNPO1	96	24	+	FCHO2	T-H	41	37	0
5	IK	14	20	+	NDUFA2	H-H	0	106	0
5	MFAP3	18	4	+	FAM114A2	H-H	0	160	0
5	LARP1	104	14	+	C5orf4	T-T	0.9	24	1
5	GTF2H2B	35	16	-	NAIP	T-H	7.8	2	0
5	LNPEP	94	19	+	LIX1	T-T	63	86	2
5	Sep-08	24	11	-	CCNI2	T-T	0	4	0
5	PPP2CA	30	7	-	SKP1	T-H	19	4	153
5	ACSL6	62	22	-	FNIP1	T-H	150	6	86
5	HMGCS1	24	11	-	NIM1	T-T	8.7	7	0
5	DROSHA	132	33	-	CDH6	T-T	71	12	0
5	FAM193B	35	12	-	DDX41	T-H	2.8	35	0
5	HNRNPA0	3	1	-	KLHL3	T-H	14	52	1
5	DHFR	24	5	-	MSH3	H-H	0	84	0
5	FAM172A	496	11	-	NR2F1	T-T	23	3109	81

5	PCDHGA1	181	5	+	DIAPH1	T-T	2	0	330
5	CNOT8	18	7	+	Gemin5	T-T	10	1	183
6	C4A	20	40	+	CYP21A2	T-H	3	1	0
6	HIST1H2AI	0.5	1	+				2	0
6	HIST1H2AH	0.5	1	+				51	0
6	KHDRBS2	608	9	-				0	84
6	HIST1H4A	0.6	1	+				0	1
6	TAF11	10	5	-	UHRF1BP1	T-T	0.3	1	0
6	ECHDC1	55	7	-	RNF146	T-T	0.15	2	2
6	BAG6	136	25	-	PRRC2A	T-T	1.2	2	4
6	NFYA	29	10	+	LOC221442	T-H	0	70	2
6	FYN	1210	13	-	NR_034110	T-H	53	6	0
6	HIST1H2BJ	0.6	1	-				7	0
6	BRD2	12	15	+	HLA-D0A	T-T	22	159	9
6	HINT3	23	5	+	TRMT11	T-H	6.2	2	12
6	ATXN1	465	4	-	GMPR	T-T	3.5	13	1
6	HIST1H1C	0.6	1	-				18	0
6	AMD1	21	9	+	GTF3C6	T-H	62	54	0
6	TMEM63B	28	24	+	MRPL14	H-H	0.2	0	98
6	HIST1H1E	0.8	1	+	HIST1H2BD	T-H	1	11/9	0/2
7	STAG3L1	8.5	10	-	PMS2L2	T-H	0.2	22	0
7	MTRNR2L6	1.4	1	+	PRSS1	T-H	81	138	0
7	RBM33	136	17	+	SHH	T-T	22	147	0
7	TWISTNB	136	5	-	TMEM196	T-H	10	1	0
7	LSM5	10	6	-	AVL9	H-H	0.3	1	0
7	NT5C3	48	11	-	FKBP9	T-T	7.1	0	1
7	TNRC18	116	27	-	SLC29A4	T-T	2.7	1	4
7	TSPAN12	70	8	+	KCND2	T-T	37	2	0
7	IRF5	12	9	+	TNPO3	T-T	4.3	5	7
7	FBXL18	38	6	-	TNRC18	T-H	52	6	1
7	ST7-AS1	1.5	1	-	CAPZA2	T-T	33	7	0
7	TTYH3	33	14	+	AMZ1	T-H	15	7	7
7	ZNF853	8.6	3	+	ZNF12	T-T	64	4	36
7	NR_038832/HOXA-.	16	8	+		bidirectional	0	0	42
7	ST7	279	21	+	ST7-AS2	H-H	0	0	3885
7	ZC3HAV1	66	13	-	ZC3HAV1L	T-H	7.5	1297	1

8	HMBOX1	165	10	+	INTS9	H-H	0.2	12	0
8	MYC	5.5	3	+	PVT1	T-H	53	36	0
8	CLN8	23	3	+	mir596		29	39	0
8	FAM160B2	15	17	+	NUDT18	T-T	2.5	44	0
8	TRPS1	260	7	-	CSMD3	T-H	1960	3	36
8	NEFL	5.7	4	-	NEFM	T-T	31	4	22
8	RAB11FIP1	40	6	-	BRF2	T-H	9.2	8	0
8	ARC	3.5	3	-	JRK	T-T	42	29	0
8	PSD3	488	16	-	NAT2	T-T	120	109	0
8	TNFRSF10D	28	9	-	TNFRSF10C	T-T	17	585	0
8	WRN	140	31	+	PURG	H-H	0	13	193
9	DNAJA1	14	9	+	SMU1	T-T	2.8	3	0
9	DMRT2	7.4	4	+	DMRT3	H-T	59	64	0
9	NCS1	64	9	+	ASS1	T-H	270	4098	0
9	TTLL11	270	9	-	mir548AA1	T-T	0	1	0
9	EXOSC3	4.9	4	-	RG9MTD3	T-T	1.3	2	0
9	PTRH1	1.7	5	-	C9orf117	T-T	0	2	0
9	PNPLA7	88	30	-	NELF	T-H	0.6	3	1
9	NOL6	12	26	-	AQP3	T-H	13	4	0
9	NINJ1	12	4	-	C9orf89	T-T	8.2	8	0
9	CAMSAP1	98	16	-	KCNT1	T-T	15	24	5
9	AGTPBP1	196	25	-	NTRK2	T-T	520	29	0
9	INPP5E	11	10	-	PMPCA	T-T	4.9	202	1
9	C9orf24	19	7	-	KIAA1161	T-H	2.1	94	2
10	TMEM236	48	4	+	MRC1	T-H	8.5	1	0
10	PWWP2B	20	3	+	C10orf91	T-H	27	1	0
10	GATA3	20	6	+	FLJ454983	H-H	1.2	3	0
10	FAM21C	66	29	+	AGAP4	T-T	31	3	0
10	PRKG1	1307	15	+	NR_038277	T-T	0	3	0
10	ZFYVE27	24	13	+	SFRP5	T-T	5.9	6	0
10	MARCH8	136	7	-	ALOX5	T-T	11	121	0
10	DDX21	28	15	+	KIAA1279	T-H	4.2	20	0
10	MORN4	19	6	-	HOGA1	T-T	1.7	1	0
10	FBXL15	3.4	4	+	CUEDC2	T-T	0.1	192	3
10	ENTPD1	165	11	+	LOC728558	T-T	0	4	0
10	KIAA1217	854	14	+	ARHGAP21	T-T	35	5	4

10	SEC24C	27	24	+	FUT11	T-H	0.1	1216	4
10	DNAJB12	22	8	-	DDIT4	T-T	56	8	1
10	FAM171A1	160	8	-	NMT2	T-H	42	32	0
10	EIF3A	45	23	-	NANOS1	T-T	0.7	33	0
10	HNRNPF	23	7	-	FXYD4	T-T	9.3	33	1
10	TIMM23	31	7	-	NCOA4	T-T	1.3	42	0
10	PYROXD2	31	16	-	HPS1	T-H	1	55	0
10	JMJD1C	300	21	-	NRBF2	T-T	12	80	0
10	AIFM2	20	9	-	H2AFY2	T-T	0	94	6
10	USMG5	8	6	-	TAF5	T-T	0	3071	0
10	C10orf118	52	16	-	MIR2110		0	207	0
10	C10orf103	28	8	+	TTC18	T-T	0	238	1
11	RASGRP2	18	19	-	NRXN2	T-H	3.7	0	1
11	PELI3	11	9	+	DPP3	T-H	3.1	1	0
11	HEPHL1	90	18	+	PANX1	T-H	14	1	0
11	RNF169	94	6	+	XRRA1	T-T	0	6	0
11	SLC35C1	9	3	+	CRY2	T-H	34	6	0
11	FAM89B	1.9	2	+	EHBP1L1	T-H	1.8	6	0
11	DRAP1	2.4	7	+	C11orf68	H-H	0.2	526	0
11	MAML2	364	5	-	MIR1260B	T-T	0	0	232
11	TMEM25	14	9	+	IFT46	T-T	0	120548	0
11	OSBP	40	15	-	OR4D9	T-T	58	1	0
11	BEST1	14	10	+	FTH1	T-T	0	0	1
11	ST3GAL4	60	11	+	KIRREL3	T-T	9	1	42
11	SIAE	41	10	-	TBRG	T-T	0	1	200
11	ADM	2.3	4	+	AMPD3	T-H	140	51	2
11	FAM160A2	23	12	-	C11orf42	T-T	0.2	3	0
11	PTPRJ	187	23	+	OR4B1	T-H	46	0	3
11	BET1L	4.6	3	-	ODF3	T-T	2.7	6	0
11	CADM1	330	10	-				7	0
11	PTGDR2	5.4	2	-	CCDC86	T-T	0	59	0
11	H2AFX	1.6	1	-	HMBS	T-T	0.3	63	0
11	HTATIP2	20	6	+	PRMT3	T-H	3.8	39	66
11	MS4A15	19	7	+	MS4A10	T-H	8.7	0	321
11	PRDM10	104	22	-	NFRKB	T-H	4.1	168	0
11	MTRNR2L8	1.3	1	-	AMPD3	T-T	0.3	708	4

12	C12orf44	7.6	4	+	KRT80	T-T	91	1	0
12	USP5	14	20	+	TPI1	T-H	0.8	2	0
12	TMBIM6	23	11	+	NCKAP5L	T-T	26	4	0
12	ARL6IP4	2.6	6	+	PITPNM	T-T	0.6	10	0
12	KNTC1	100	54	+	RSRC2	H-H	0.25	11	0
12	PUS1	14	6	+	EP400	T-H	6.1	41	0
12	LRRC43	36	16	+	B3GNT4	T-H	0.2	0	1
12	CAPRIN2	45	18	-	IPO8	T-H	13	1	2
12	PCBP2	28	15	+	MAP3K12	T-T	0	7	1
12	ADCY6	22	22	-	LOC255411	T-T	0.4	1	23
12	KDM5A	109	28	-	SLC6A13	T-H	17	5	0
12	ATP5G2	11	5	-	ATF7	T-H	38	5	0
12	PUS7L	30	9	-	IRAK4	H-H	0.15	6	0
12	HIST4H4	0.6	1	-	H2AFJ	H-H	3.1	10	1
12	C12orf51	225	56	-	TRAFD1	T-T	6.6	20	0
12	MIP	5.2	4	-	TIMELESS	T-H	0.1	22	1
12	KCTD10	28	7	-	MYO1H	T-T	0.3	900	0
12	MED13L	320	25	-				346	0
12	RPLP0	4.5	8	-	NR_038924	H-H	0.1	264	0
13	FLT3	97	21	-	PRHOXNB	T-H	15	0	10
13	ZMYM2	130	23	+	GJA3	T-T	46	9288	0
13	CKAP2	21	9	+	TPTE2P3	T-H	13	5	1
13	FAM48A	50	25	-	EXOSC8	T-T	0	2	160
13	DIS3	27	22	-	BORA/PIBF1	T-T/H-H	0/0	14	4
13	DOCK9	292	36	-	SLC15A1	T-H	40	61	0
13	ALG5	49	10	-	EXOSC8	H-H	1.2	486	0
14	SPTB	77	33	-	PLEKHG3	T-T	1.9	1	12
14	GMPR2	56	9	+	TINF2	T-T	0.4	0	4
14	FAM179B	110	19	+	KLHL28	H-H	0.3	0	9
14	BTBD7	96	12	-	C14orf142	T-T	8.4	10	0
14	HECTD1	107	36	-	AP4S1	T-T	3.7	10	0
14	GCH1	60	6	-	SAMD4A	T-T	48	10	1735
14	SLC25A47	7.1	6	+	WARS	T-T	3.4	0	15
14	TGM1	14	15	-	TINF2	T-H	6.4	695	0
15	PAQR5	106	9	+	KIF23	T-H	6.7	162	0
15	AKAP13	370	29	+	KLHL25	T-T	10	9484	0

15	SHF	34	8	-	DUOX1	T-T	1.6	1	9335
15	CHD2	127	38	+	RGMA	T-T	15	5	2
15	ZNF280D	100	17	-	MNS1	T-H	160	3	0
15	TLN2	196	54	+	MGC15885	H-H	2.1	0	6
15	SMAD6	80	6	+	SMAD3	T-H	280	7	7
15	C15orf48	2.9	4	+	MIR147B			111/34	
16	PDP2	10	2	+	CDH16	T-T	17	2	0
16	CDK10	9.4	13	+	SPATA2L	T-T	0	2	0
16	NFAT5	136	14	+	NQO1	T-T	5	105	0
16	MIR1538	0.6	14	-	NFAT5	H-H	0	190	0
16	NDE1	58	10	+	KIAA0430	H-H	0.1	13244	1
16	SF3B3	53	28	+	COG4	H-H	0.2	171	1
16	CASKIN1	18	20	-	TRAF7	T-T	0	2	2
16	SRRM2	18	15	+	TCEB2	T-T	0	50	2
16	SRL	52	6	-	ADCY9	T-H	75	3	0
16	HCFC1R1	1.6	4	-	TNFRSF12A	T-T	0.25	4	0
16	JMJD8	2.7	9	-	STUB1	T-T	0	6	253
16	MAZ	4.7	6	+	PRRT2	T-H	1	82	10
16	KIAA0182	65	16	-	GINS2	T-T	1.5	12	8
16	KIFC3	45	20	-	KATNB1	T-T	1	14	39
16	VPS4A	13	11	+	PDF	T-T	3.6	4	17
16	C16orf58	19	13	-	SLC5A2	T-T	0	43	0
16	FTSJD1	7.4	3	-	HYDIN	T-H	50	102	1
16	FBXO31	63	11	-	C16orf95	T-H	12	123	0
16	ACD	3.3	12	-	RLTPR	T-T	0	296	3
16	CTU2	9	15	+	PIEZO1	T-T	0	0	1724
17	ATP5G1	3.2	5	+	UBE2Z	T-H	12	1	0
17	SGSM2	44	22	+	TSR1	H-H	0.1	6	0
17	ATP6V0A1	64	22	+	NAGLU	T-H	13	15	0
17	MRPL12	4.2	5	+	SLC25A10	T-H	4.8	28	0
17	EIF4A1	6.7	11	+	SENP3/CD68	H-T/T-H	0/0.5	73	0
17	ALOX12B	15	15	-	ALOX15B	T-T	23	0	103
17	KCTD11	3.1	1	+	TNEM95	T-H	0.25	0	1
17	SPNS2	39	13	+	MYBBP1A	T-T	0	1	1
17	TMUB2	4.8	4	+	ATXN7L3	T-T	0.1	26	1
17	MAP2K4	120	12	+	ZNF18	H-H	23	69	1

17	HS3ST3B1	45	3	+	CDRT15	H-H	64	142	1
17	MTRNR2L1	1.6	1	+	FLJ36000	H-T	110	552	1
17	SLC9A3R1	21	6	+	MIR3615		0	204	1
17	SEZ6	50	16	-	PHF12	T-H	4.1	2	0
17	ZNF207	20	12	+	MIR632		0	0	2
17	ARRB2	11	15	+	MED11	T-H	10	4	2
17	FTSJ3	8.3	21	-	DDX42/PSMC5	T-T/H-H	0.15/0	2	10
17	FAM104A	25	4	-	COG1	T-T	0	6	0
17	ANAPC11	8.9	4	+	ALYREF	H-H	0.15	10	3
17	HOXB8	2.6	2	-	HOXB7	T-H	1.3	3	118
17	HES7	3.5	4	-	ALOXE3	T-H	1.7	4	126
17	ATXN7L3	6.4	12	-	TMUB2	T-T	0.1	7	0
17	GPATCH8	110	8	-	ITGA2B	T-H	5.8	8	0
17	CCDC103	3.5	4	-	EFTUD2/GFAP	H-H/T-T	0.2/2.4	8	0
17	KCNJ12	44	4	+	C17orf51	T-T	107	2	12
17	HN1	19	6	-	NT5C	T-H	3.5	16	2
17	GGA3	25	17	-	NUP85/MRPS7	T-T/H-H	0.8/0	25	0
17	FASN	20	43	-	DUS1L	T-H	12	26	0
17	LOC404266	16	7	+	HOXB7	T-T	0.8	1	53
17	DDX5	8.1	14	-	CEP95/POLG2	H-H/T-H	0.7/1.3	83	0
17	SRSF1	6.5	3	-	VEZF1	T-H	13	93	4
17	TMEM107	3.5	5	-	VAMP2	T-H	10	540	0
17	CTC1	23	23	-	LINC00324	T-H	0.8	1435	0
17	NCOR1	182	40	-	TTC19	T-T	0.7	280	2
18	CCDC165	112	16	+	NDUFV2	T-H	269	1	0
18	INO80C	30	7	-	ZNF396	T-H	92	0	1
18	PMAIP1	5.4	2	+	CCBE1	H-H	203	29	0
18	RPL17	5.5	7	-	DYM	T-H	20	1469	0
19	ZSCAN22	15	3	+	A1BG	T-T	4.5	2	0
19	TMEM91	20	5	+	B9D2	H-H	0	5	0
19	ZNF264	31	4	+	AURKC	T-H	8.2	17	0
19	EMR2	48	20	-	ZNF333	T-T	11	0	21
19	PDE4C	40	17	-	RAB3A	T-H	4	0	30
19	ZNF419	7	4	+	ZNF773	T-H	5.3	30	0
19	SUPT5H	31	29	+	TIMM50	T-H	3.8	37	0
19	ZNF763	15	4	+	ZNF433	T-T	34	58	0

19	ZNF507	42	7	+	DPY19L3	T-H	18	102	0
19	ZIM2	65	10	-	MIMT1	H-H	0.2	0	573
19	EPS15L1	110	22	-	KLF2	T-T	34	1	0
19	ZNF98	31	4	-	ZNF729	T-T	74	1	0
19	CRTC1	99	15	+	COMP	T-T	0.4	3	1
19	UPF1	36	24	+	GDF1	T-T	0.3	15	1
19	KISS1R	3.8	5	+	ARID3A	T-H	5	840	1
19	CACNG8 MIR935)	27	4	+	CACNG6	T-H	2.1	116	2
19	LYPD3	5	5	-	TEX101	T-T	42	4	26247
19	ATP13A1	18	26	-	GIMP	T-H	1.5	5	0
19	AKT1S1	8.5	5	-	TBC1D17	H-H	0.04	7	1
19	MAU2	37	19	+	SUGP1	H-H	0.3	3	14
19	RABAC1	2.7	5	-	ATP1A3	H-T	7.3	22	29
19	MUM1	23	15	+	NDUFS7	T-H	5.5	1	35
19	TNPO2	25	28	-	FBXW9	T-H	2.5	49	1
19	EPN1	20	13	+	NLRP9/U2AF2	T-T/H-T	12/0.5	74	90
19	ITPKC	23	7	+	C19orf54/ADCK4	T-T/H-H	0/0.2	3	110
19	ZNF850	29	5	-	ZNF567	T-T	21	120	0
20	RAB22A	57	6	+	PPP4R1L	H-H	0.3	184	0
20	RAE1	27	14	+	RBM38	T-H	13	0	35
20	PRIC285	16	20	-	C20orf195	T-T	1.4	51	0
21	ADAMTS1	9.3	9	-	ADAMTS5	H-T	73	29	0
22	DERL3	5	7	-	SMARCB1	T-T	0	0	2
22	INPP5J	11	13	+	PLA2G3	T-T	0.1	2	0
22	MGAT3	35	2	+	SMCR7L	T-H	10	3	0
22	SGSM1	120	23	+	TMEM211	T-T	8.4	4	0
22	SMTN	23	21	+	SELM	T-T	0.15	14	0
22	CCDC134	25	7	+	MEI1/SREBF2	H-T/T-H	1.2/6.8	25	0
22	TNRC6B	290	21	+	ASDL	T-H	10	70	0
22	FAM109B	5.2	3	+	C22orf32	T-H	0.25	0	1
22	YPEL1	40	5	-	PPIL2	T-T	0	2	0
22	THOC5	45	21	-	NEFH	T-T	16	6	0
22	CENPM	8.5	7	-	TNFRSF13C/BK25(	T-H/H-H	5/12	4	0
22	ACO2	60	15	+	PHF5A/POLR3H	H-H/T-T	0.4/0	2	4
22	DGCR8	31	14	+	TRMT2A	T-T	0	244	6
22	CYP2D6	4.5	9	-	NR_034118	T-T	1.1	6	0

22	DDX17	23	13	-	KDEL	T-T	0	15	0
22	PLA2G6	70	17	-		T-H	0.8	71	1
X	TBL1X	255	12	+	GPR143	T-T	5.7	3	0
X	PDZD4	28	8	-	SSR4	T-T	3.8	0	540
X	WNK3	160	21	-	FAM120C	T-H	9.5	1	1
X	SAT1	3.2	7	+	APOO	T-T	47	142	1
X	MAGED4B	7.6	13	+	XAGE2	T-H	176	5	2
X	BCAP31	23	8	-	ABCD1/SLC6A8	H-H/T-T	0.1/4	3	2
X	MID1IP1	5.2	3	+	TSPAN7	H-T	117	5	4
X	SMC1A	48	25	-	RIBC1	H-H	0.2	5	2
X	GAGE10	16	5	+	GAGE12J	T-H	2.1	0	6
X	DDX3X	17	17	+	NYX	T-H	97	150	6
X	MTRNR2L10	1.1	1	-	FAM104B	T-H	20	7	0
X	MID1	388	11	-	CLCN4	T-T	200	9	0
X	FGF13	575	7	-	LOC158696	T-H	14	9	0
X	Cxorf49	4	6	-	Cxorf49/NR_03400	H-T/T-T	44/11	12	0
X	Cxorf49	4	6	+	Cxorf49	T-H	44	0	16
X	IRS4	3.9	1	-	COL4A5	T-T	35	32	1
X	KLHL15	43	4	-	Cxorf58	T-T	43	327	0
