# Supplementary material for: Contribution of natural antisense transcription to an endogenous siRNA signature in human cells
Source: BMC Genomics. 2014 Jan 13;15:19. doi: 10.1186/1471-2164-15-19 (PMC3898206; doi:10.1186/1471-2164-15-19)
Supplement: Additional file 2: Figure S1 — Genes with short reads compared to the total number of genes. [file 1471-2164-15-19-S2.pdf]

Genes with short reads compared to the total number of genes

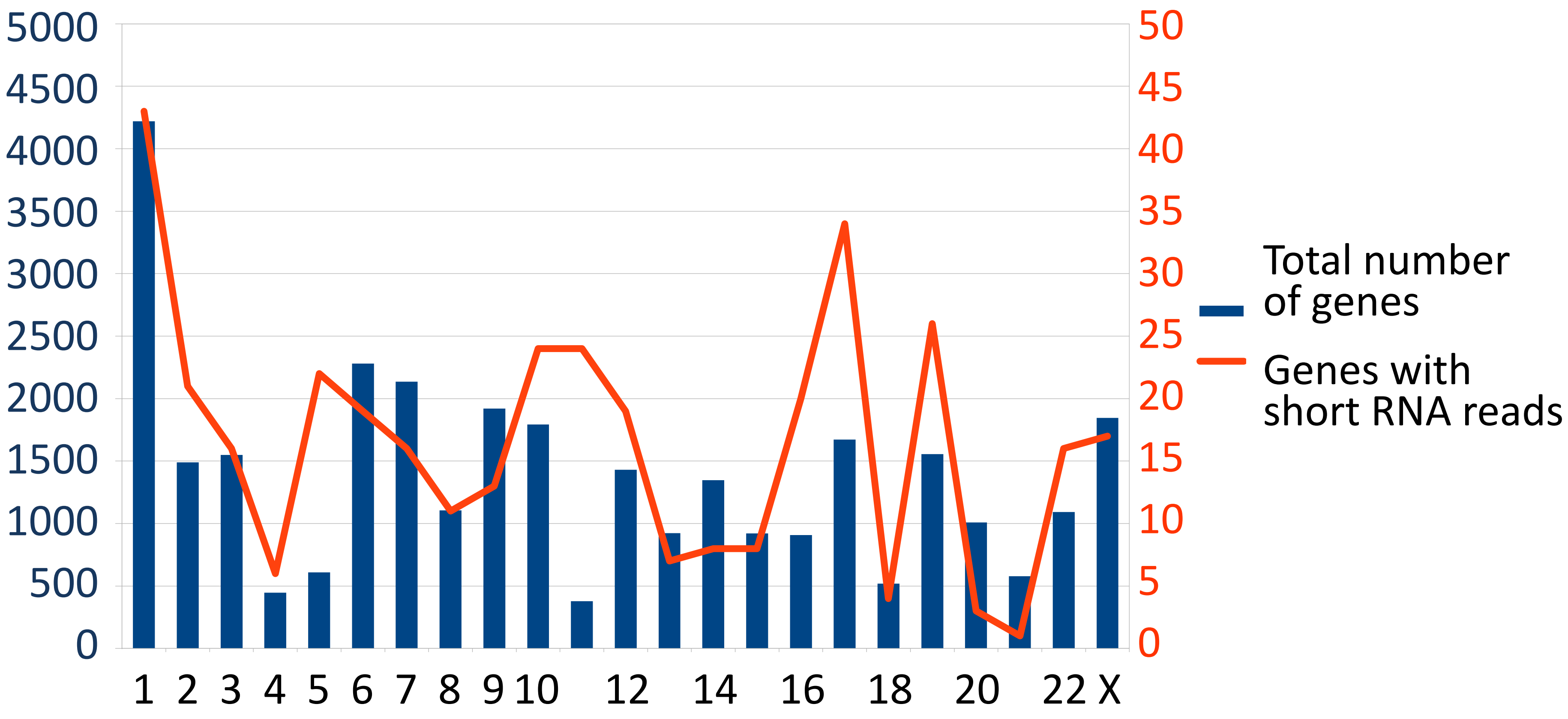

The x-axis represents the 23 human chromosomes excluding the Y chromosome which is not present in HEK293 cells. The left y-axis (blue) represents the approximate number of genes on the respective chromosomes. The right y-axis (orange) represents the number of genes that produce short RNAs.
