# Supplementary material for: Contribution of natural antisense transcription to an endogenous siRNA signature in human cells
Source: BMC Genomics. 2014 Jan 13;15:19. doi: 10.1186/1471-2164-15-19 (PMC3898206; doi:10.1186/1471-2164-15-19)
Supplement: Additional file 3: Figure S2 — Expression of endo-siRNA related genes. [file 1471-2164-15-19-S3.pdf]

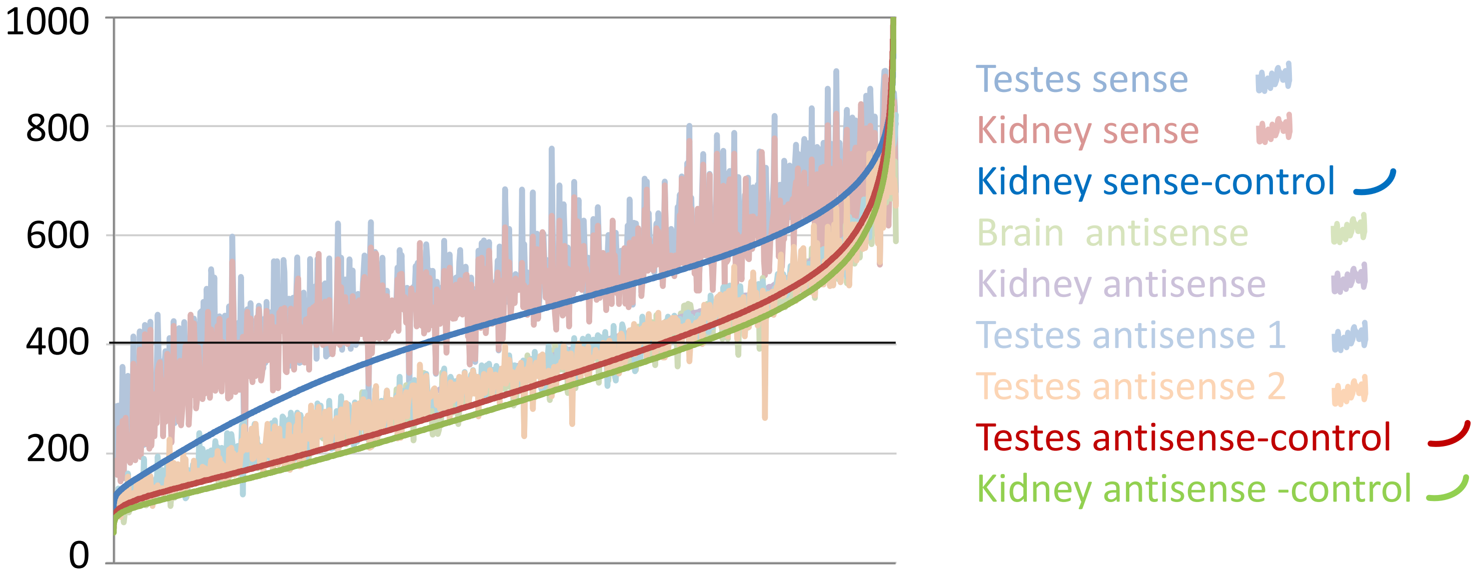

Expression of the endo-siRNA related genes compared to average gene expression: Order of genes kept the same as “Kidney antisense” which were sorted from lowest to highest expression.
