# Supplementary material for: Contribution of natural antisense transcription to an endogenous siRNA signature in human cells
Source: BMC Genomics. 2014 Jan 13;15:19. doi: 10.1186/1471-2164-15-19 (PMC3898206; doi:10.1186/1471-2164-15-19)
Supplement: Additional file 4: Table S2 — Expression data of miRNAs and structural RNAs. [file 1471-2164-15-19-S4.pdf]

**MicroRNAs**						**Structural short RNAs**					
Name	wild type reads	C5	C12	C5 normalized reads	C12	Name	wild type reads	C5	C12	C5 normalized reads	C12
hsa-let-7a-1	77523	180363	256290	80550.7	81612.7	ACA13	471	211	264	255.2	362.9
hsa-let-7a-2	9958	16766	23869	7487.7	7600.8	ACA17	311	86	76	104.0	104.5
hsa-let-7a-3	79912	183394	262769	81904.3	83675.8	ACA18	142	466	115	563.6	158.1
hsa-let-7b	893	2224	4060	993.2	1292.9	ACA23	112	51	32	61.7	44.0
hsa-let-7c	19553	47075	75157	21023.8	23932.9	ACA24	285	65	116	78.6	159.5
hsa-let-7d	489	1100	1505	491.3	479.3	ACA26	1131	219	235	264.9	323.1
hsa-let-7e	4677	8902	9752	3975.7	3105.4	ACA28	106	80	126	96.8	173.2
hsa-let-7f-1	72548	168253	181187	75142.3	57697.0	ACA3	543	109	136	131.8	187.0
hsa-let-7f-2	23026	44026	55734	19662.1	17747.9	ACA31	430	73	63	181.4	281.8
hsa-let-7g	7420	12480	19246	5573.6	6128.7	ACA3-2	111	150	205	88.3	86.6
hsa-let-7i	4986	13618	18035	6081.8	5743.0	ACA33	110	55	65	66.5	89.4
hsa-mir-100	198	429	659	191.6	209.9	ACA34	434	6324	2055	7648.2	2825.1
hsa-mir-101-1	1500	5320	7247	2375.9	2307.7	ACA42	919	144	152	174.2	209.0
hsa-mir-101-2	1871	6650	7982	2969.9	2541.8	ACA43	1778	381	477	460.8	655.8
hsa-mir-103-1	8121	11159	15720	4983.6	5005.9	ACA44	791	317	296	383.4	406.9
hsa-mir-103-1-a	8105	11156	15715	4982.3	5004.3	ACA45	8457	22203	29220	26852.3	40170.3
hsa-mir-103-2	52958	97550	130916	43566.1	41688.7	ACA47	276	154	154	186.2	211.7
hsa-mir-103-2-a	52810	97281	130642	43446.0	41601.5	ACA48	441	311	483	376.1	664.0
hsa-mir-106b	6412	16764	22480	7486.9	7158.5	ACA50	170	210	252	254.0	346.4
hsa-mir-107	6025	9238	12481	4125.7	3974.4	ACA54	279	285	472	344.7	648.9
hsa-mir-10a	3420845	8302635	11968561	3707982.5	3811254.2	ACA56	165	65	110	78.6	151.2
hsa-mir-10b	1076421	2906801	4249191	1298186.3	1353107.3	ACA57	1813	402	738	486.2	1014.6
hsa-mir-1180	809	2342	3122	1045.9	994.2	ACA58	360	153	178	185.0	244.7
hsa-mir-1201	7503	9774	5510	4365.1	1754.6	ACA5c	143	23	54	27.8	74.2
hsa-mir-1226	230	347	528	155.0	168.1	ACA61	558	215	226	260.0	310.7
hsa-mir-1254	190	211	371	94.2	118.1	ACA63	175	239	278	289.0	382.2

hsa-mir-1255a	1033	1794	1999	801.2	636.6	ACA65	424	31	53	37.5	72.9
hsa-mir-1257	112	193	199	86.2	63.4	ACA7	12473	3026	2240	3659.6	3079.4
hsa-mir-125a	39100	70865	105683	31648.5	33653.6	ACA7B	32963	1573	3206	1902.4	4407.5
hsa-mir-125b-1	350	647	764	289.0	243.3	ACA9	239	46	56	55.6	77.0
hsa-mir-125b-2	9322	15302	20435	6833.9	6507.3	E2	1100	563	859	680.9	1180.9
hsa-mir-126	3413	8949	12309	3996.7	3919.7	E3	245	86	88	104.0	121.0
hsa-mir-1262	179	278	249	124.2	79.3	HBI-100	647	1490	1787	1802.0	2456.7
hsa-mir-1266	851	1392	1871	621.7	595.8	HBI-43	10436	12346	16282	14931.2	22383.7
hsa-mir-1271	668	528	875	235.8	278.6	HBI-6	170	89	125	107.6	171.8
hsa-mir-1272	228	198	285	88.4	90.8	HBII-108B	43581	10063	14178	12170.2	19491.3
hsa-mir-1274b	149	416	429	185.8	136.6	HBII-135	503	833	442	1007.4	607.6
hsa-mir-1276	418	658	886	293.9	282.1	HBII-142	5482	5386	7783	6513.8	10699.7
hsa-mir-128-1	3922	7148	9766	3192.3	3109.9	HBII-166	239	50	44	60.5	60.5
hsa-mir-128-2	2377	5171	6664	2309.4	2122.1	HBII-180A	3433	2276	2501	2752.6	3438.3
hsa-mir-1286	389	2237	2910	999.1	926.7	HBII-180B	240735	21037	42123	25442.1	57908.7
hsa-mir-1291	394	6315	2048	2820.3	652.2	HBII-180C	39180	58521	16638	70775.2	22873.1
hsa-mir-1293	275	231	379	103.2	120.7	HBII-202	1553	1834	1779	2218.0	2445.7
hsa-mir-1296	1254	1286	1960	574.3	624.1	HBII-210	32448	19319	19564	23364.4	26895.7
hsa-mir-1301	953	1163	2138	519.4	680.8	HBII-234	5996	6785	9178	8205.8	12617.5
hsa-mir-1303	330	271	371	121.0	118.1	HBII-239	609	809	694	978.4	954.1
hsa-mir-1304	1130	1999	2584	892.8	822.8	HBII-240	7529	4093	5333	4950.1	7331.6
hsa-mir-1306	111	210	350	93.8	111.5	HBII-251	8240	7901	11262	9555.5	15482.5
hsa-mir-1307	3091	11571	16677	5167.6	5310.6	HBII-276	189	137	135	165.7	185.6
hsa-mir-1308	185	168	219	75.0	69.7	HBII-289	280	115	152	139.1	209.0
hsa-mir-130a	5371	12610	16531	5631.7	5264.1	HBII-295	165	155	117	187.5	160.8
hsa-mir-130b	29337	50549	70115	22575.3	22327.3	HBII-296A	4938	2420	2865	2926.7	3938.7
hsa-mir-132	1577	3922	6051	1751.6	1926.9	HBII-296B	2118	785	1039	949.4	1428.4
hsa-mir-133a-2	168	407	719	181.8	229.0	HBII-316	4120	772	1095	933.7	1505.4
hsa-mir-138-1	470	688	953	307.3	303.5	HBII-336	55571	56208	93876	67977.9	129056.4

hsa-mir-138-2	124	384	393	171.5	125.1	HBII-382	7832	4244	6952	5132.7	9557.3
hsa-mir-140	17340	29592	38376	13215.9	12220.4	HBII-419	11945	4310	5473	5212.5	7524.0
hsa-mir-141	89	429	672	191.6	214.0	HBII-420	2250	41990	10506	50782.6	14443.2
hsa-mir-1468	308	1278	2455	570.8	781.8	HBII-429	25427	9250	13224	11186.9	18179.7
hsa-mir-146a	448	853	1389	381.0	442.3	HBII-55	4147	6173	4782	7465.6	6574.1
hsa-mir-146b	83172	228660	260408	102120.3	82924.0	HBII-82	509	744	333	899.8	457.8
hsa-mir-147b	102	195	177	87.1	56.4	HBII-82B	14710	6252	7878	7561.2	10830.3
hsa-mir-148a	254009	794604	1179384	354872.6	375561.6	HBII-95	1600	1086	1233	1313.4	1695.1
hsa-mir-148b	32289	64786	102300	28933.6	32576.3	HBII-95B	406	105	62	127.0	85.2
hsa-mir-149	809	1270	2119	567.2	674.8	HBII-99	4336	13378	4953	16179.3	6809.2
hsa-mir-151	59082	132083	181588	58988.7	57824.7	HBII-99B	87247	7702	3914	9314.8	5380.8
hsa-mir-152	802	2058	2815	919.1	896.4	hTR	909	107	115	129.4	158.1
hsa-mir-15a	857	2870	3817	1281.8	1215.5	mgh18S-12	7564	3190	3082	3858.0	4237.0
hsa-mir-15b	10969	21005	29112	9380.9	9270.4	mgh28S-24	1909	2434	1587	2943.7	2181.7
hsa-mir-16-1	1500	4227	5660	1887.8	1802.4	mgh28S-24	2785	1579	1060	1909.6	1457.2
hsa-mir-16-2	4061	8536	11407	3812.2	3632.4	mgU12-22,	304	67	95	81.0	130.6
hsa-mir-17	12555	18663	26183	8335.0	8337.7	mgU2-19/3	82497	76593	131048	92631.4	180158.7
hsa-mir-181a-1	10183	14558	18832	6501.6	5996.8	mgU2-25/6	8029	4258	6959	5149.6	9566.9
hsa-mir-181a-2	104351	150235	215249	67095.4	68543.6	mgU6-47	1294	1145	1524	1384.8	2095.1
hsa-mir-181b-1	6938	9025	12749	4030.6	4059.8	mgU6-53	161	716	322	865.9	442.7
hsa-mir-181b-2	33037	44940	61749	20070.3	19663.3	mgU6-77	995	637	652	770.4	896.3
hsa-mir-181c	17051	35446	44245	15830.3	14089.3	SNORD119	20247	8234	10788	9958.2	14830.8
hsa-mir-181d	8989	23356	29079	10430.9	9259.9	SNORD121	862	313	263	378.5	361.6
hsa-mir-182	550275	751719	890750	335720.0	283649.4	SNORD121	434	382	347	462.0	477.0
hsa-mir-1826	3769	11933	18418	5329.3	5865.0	SNORD124	328	1264	449	1528.7	617.3
hsa-mir-183	85174	197827	368898	88350.2	117471.4	SNORD125	152	60	45	72.6	61.9
hsa-mir-185	2049	3612	4892	1613.1	1557.8	SNORD126	7503	9774	5510	11820.7	7574.9
hsa-mir-186	181508	276683	339529	123567.5	108119.2	SNORD127	386	3629	1216	4388.9	1671.7
hsa-mir-187	107	144	177	64.3	56.4	snR38A	3334	2005	3031	2424.8	4166.9

hsa-mir-18a	1247	2604	3500	1163.0	1114.5	snR38B	31884	11262	17924	13620.2	24641.1
hsa-mir-18b	853	1802	2433	804.8	774.8	snR38C	2185	1729	2278	2091.0	3131.7
hsa-mir-191	266627	467801	673252	208921.4	214389.6	snR39B	85111	66317	28353	80203.7	38978.4
hsa-mir-1910	176	551	484	246.1	154.1	U100	351	104	108	125.8	148.5
hsa-mir-192	37279	102012	134044	45558.9	42684.8	U101	8776	18596	5894	22490.0	8102.8
hsa-mir-193b	2768	4078	5449	1821.2	1735.2	U102	2445	23779	6516	28758.3	8957.9
hsa-mir-194-1	272	580	758	259.0	241.4	U103	129	277	88	335.0	121.0
hsa-mir-194-2	589	1068	1412	477.0	449.6	U103B	153	271	92	327.7	126.5
hsa-mir-195	284	784	1120	350.1	356.7	U104	19628	36162	22395	43734.3	30787.6
hsa-mir-196a-1	13158	28667	37223	12802.8	11853.2	U105	1164	517	690	625.3	948.6
hsa-mir-196a-2	23701	57942	71376	25877.1	22728.9	U105B	477	4135	1784	5000.9	2452.6
hsa-mir-196b	36645	89609	121845	40019.7	38800.2	U106	26336	2047	1041	2475.6	1431.1
hsa-mir-197	835	1460	2133	652.0	679.2	U107	156	25	39	30.2	53.6
hsa-mir-1974	24704	8017	6050	3580.4	1926.6	U13	6902	3170	4108	3833.8	5647.5
hsa-mir-1975	31645	25347	30217	11320.0	9622.3	U15A	60950	51126	69723	61831.7	95851.9
hsa-mir-1977	5918	2133	2788	952.6	887.8	U16	162	564	262	682.1	360.2
hsa-mir-1978	3822	5450	5123	2434.0	1631.4	U17a	2339	326	487	394.3	669.5
hsa-mir-19a	3754	10800	14550	4823.3	4633.3	U17b	1374	166	270	200.8	371.2
hsa-mir-19b-1	6762	13293	17940	5936.7	5712.8	U18A	22656	35603	20863	43058.2	28681.5
hsa-mir-19b-2	1310	1917	2840	856.1	904.4	U18B	5366	5610	3051	6784.7	4194.4
hsa-mir-200c	199	226	287	100.9	91.4	U18C	4816	1687	1528	2040.3	2100.6
hsa-mir-204	737	1166	1968	520.7	626.7	U19	5443	5207	7266	6297.3	9989.0
hsa-mir-20a	4161	7879	12931	3518.8	4117.7	U19-2	145	35	41	42.3	56.4
hsa-mir-20b	230	341	662	152.3	210.8	U20	46828	12772	20468	15446.4	28138.5
hsa-mir-21	14555	34102	48408	15230.1	15415.0	U21	5781	5921	6763	7160.8	9297.5
hsa-mir-210	153	704	868	314.4	276.4	U22	281	241	239	291.5	328.6
hsa-mir-2110	207	340	383	151.8	122.0	U24	16958	2383	2668	2882.0	3667.8
hsa-mir-212	380	657	1077	293.4	343.0	U25	37656	8766	9478	10601.6	13029.9
hsa-mir-215	918	1704	2265	761.0	721.3	U26	19389	8825	12745	10672.9	17521.2

hsa-mir-218-1	6077	20355	27133	9090.6	8640.2	U27	3623	5680	5661	6869.4	7782.5
hsa-mir-218-2	1849	4507	6579	2012.8	2095.0	U28	494	366	427	442.6	587.0
hsa-mir-219-1	192	382	454	170.6	144.6	U29	25158	145331	55158	175763.0	75828.7
hsa-mir-22	27566	58990	72957	26345.1	23232.3	U3	8893	6873	7218	8312.2	9923.0
hsa-mir-221	6636	15567	16205	6952.3	5160.3	U30	74868	3838	4870	137140.9	205505.0
hsa-mir-222	15865	40565	29859	18116.5	9508.3	U31	7057	190	230	8124.7	9516.0
hsa-mir-23a	759	1353	1796	604.3	571.9	U3-2	3618	520	428	4641.7	6695.0
hsa-mir-23b	3059	4029	6356	1799.4	2024.0	U32A	1678	160	264	4114.4	2341.2
hsa-mir-24-1	1237	3462	4809	1546.1	1531.4	U3-2B	627	113396	149485	229.8	316.2
hsa-mir-24-2	672	1199	1978	535.5	629.9	U33	2364	6718	6922	9422.4	10664.0
hsa-mir-25	170524	478447	521995	213675.9	166223.5	U3-3	1584	3402	1703	628.9	588.4
hsa-mir-26a-1	156967	443079	668794	197880.5	212970.0	U34	376	7791	7757	166.9	154.0
hsa-mir-26a-2	14713	32833	47371	14663.3	15084.8	U3-4	666	138	112	193.5	362.9
hsa-mir-26b	16048	46118	61751	20596.4	19663.9	U35A	135	27	32	32.7	44.0
hsa-mir-27a	7212	23603	30843	10541.2	9821.6	U35B	551	108	165	130.6	226.8
hsa-mir-27b	69578	250621	340845	111928.1	108538.3	U36A	2488	2541	4252	3073.1	5845.5
hsa-mir-28	14373	26372	34442	11777.8	10967.7	U36B	73792	21331	35794	25797.7	49207.9
hsa-mir-296	412	548	844	244.7	268.8	U36C	929	636	505	769.2	694.3
hsa-mir-29a	1523	3758	4860	1678.3	1547.6	U37	4912	7350	8869	8889.1	12192.7
hsa-mir-29c	283	555	695	247.9	221.3	U38A	286562	37643	55642	45525.4	76494.0
hsa-mir-301a	3861	12669	17177	5658.0	5469.8	U38B	3371	1638	2187	1981.0	3006.6
hsa-mir-301b	1672	3539	5381	1580.5	1713.5	U41	469	77	92	93.1	126.5
hsa-mir-30a	196448	419603	537959	187396.0	171307.0	U42A	11283	96650	42017	116888.3	57763.0
hsa-mir-30b	8778	16643	25321	7432.8	8063.2	U42B	730	1877	1004	2270.0	1380.3
hsa-mir-30c-1	62355	73009	95661	32606.0	30462.2	U43	13287	347724	119846	420536.8	164758.7
hsa-mir-30c-2	4958	5603	7243	2502.3	2306.5	U44	141466	175354	226220	212072.8	310996.7
hsa-mir-30d	136814	209894	275996	93739.3	87887.8	U45A	32214	5880	2955	7111.3	4062.4
hsa-mir-30e	152128	378943	525596	169237.1	167370.2	U45B	1152	512	891	619.2	1224.9
hsa-mir-31	8096	13351	18080	5962.6	5757.4	U45C	1888	6799	2942	8222.7	4044.5

hsa-mir-32	137	505	687	225.5	218.8	U46	1027	3917	1408	4737.2	1935.7
hsa-mir-320a	6196	10321	15163	4609.4	4828.5	U47	4804	878	1126	1061.9	1548.0
hsa-mir-320b-1	1225	3392	4072	1514.9	1296.7	U48	1557	8408	2826	10168.6	3885.1
hsa-mir-320b-2	775	1318	1779	588.6	566.5	U49A	4267	58769	16360	71075.1	22491.0
hsa-mir-324	284	586	720	261.7	229.3	U49B	544	1082	414	1308.6	569.1
hsa-mir-328	174	418	570	186.7	181.5	U50	75925	64160	83738	77595.0	115119.1
hsa-mir-330	1729	3985	5021	1779.7	1598.9	U50B	13832	1773	2395	2144.3	3292.5
hsa-mir-331	1661	3469	4938	1549.3	1572.5	U51	2489	1008	1244	1219.1	1710.2
hsa-mir-338	1564	3917	5788	1749.3	1843.1	U52	2412	6725	6438	8133.2	8850.7
hsa-mir-339	1213	3005	3841	1342.0	1223.1	U53	879	179	262	216.5	360.2
hsa-mir-33a	273	156	212	69.7	67.5	U54	154	61	160	73.8	220.0
hsa-mir-33b	113	113	163	50.5	51.9	U55	264	829	648	1002.6	890.8
hsa-mir-340	13677	50225	59801	22430.6	19043.0	U56	374	1596	700	1930.2	962.3
hsa-mir-342	6691	10104	13064	4512.5	4160.1	U57	27453	14204	20523	17178.3	28214.1
hsa-mir-345	5031	11075	13511	4946.1	4302.4	U58A	1538	3546	2461	4288.5	3383.3
hsa-mir-34a	1632	5851	8723	2613.1	2777.7	U58B	5074	7520	6042	9094.7	8306.3
hsa-mir-361	8220	14383	20152	6423.5	6417.2	U58C	96408	104253	133951	126083.4	184149.6
hsa-mir-362	1386	1680	3534	750.3	1125.4	U59A	1422	734	693	887.7	952.7
hsa-mir-363	1285	5133	5232	2292.4	1666.1	U59B	4800	2265	1850	2739.3	2543.3
hsa-mir-365-1	812	1506	1636	672.6	521.0	U60	3315	10289	4860	12443.5	6681.3
hsa-mir-365-2	886	1607	1788	717.7	569.4	U61	4649	4709	2540	5695.1	3491.9
hsa-mir-374a	2677	8896	12560	3973.0	3999.6	U62A	45857	12415	17201	15014.7	23647.1
hsa-mir-374b	4573	8762	13189	3913.1	4199.9	U62B	45411	12010	16923	14524.9	23265.0
hsa-mir-375	1366	4168	5515	1861.4	1756.2	U63	8879	2493	2584	3015.0	3552.4
hsa-mir-378	83488	157879	223997	70509.3	71329.3	U64	2634	7955	1858	9620.8	2554.3
hsa-mir-421	18650	34490	44382	15403.3	14133.0	U65	1539	817	472	988.1	648.9
hsa-mir-423	163950	258706	318418	115538.9	101396.6	U66	121	52	54	62.9	74.2
hsa-mir-424	355	1068	1253	477.0	399.0	U67	123	120	58	145.1	79.7
hsa-mir-425	10761	18035	22771	8054.5	7251.2	U68	63	1770	290	2140.6	398.7

hsa-mir-449a	272	1010	1415	451.1	450.6	U69	415	134	192	162.1	264.0
hsa-mir-450a-1	159	386	534	172.4	170.0	U70	130	490	165	592.6	226.8
hsa-mir-450a-2	151	337	454	150.5	144.6	U71a	370	207	298	250.3	409.7
hsa-mir-450b	1187	3347	4526	1494.8	1441.3	U71b	257	49	76	59.3	104.5
hsa-mir-454	7169	8681	12044	3877.0	3835.3	U71d	10440	1923	1974	2325.7	2713.8
hsa-mir-455	309	632	874	282.3	278.3	U73a	342	268	124	324.1	170.5
hsa-mir-484	13246	22543	25921	10067.8	8254.3	U74	5810	9335	9152	11289.7	12581.7
hsa-mir-486	7641	55590	78633	24826.7	25039.8	U75	3931	4252	1593	5142.4	2190.0
hsa-mir-488	283	1445	2134	645.3	679.5	U76	40267	4417	5382	5341.9	7398.9
hsa-mir-497	205	588	865	262.6	275.4	U77	413	708	962	856.3	1322.5
hsa-mir-500	11307	16164	25082	7218.9	7987.1	U78	33878	28189	31294	34091.7	43021.5
hsa-mir-501	2279	2121	3611	947.2	1149.9	U79	826	244	183	295.1	251.6
hsa-mir-502	569	800	1256	357.3	400.0	U8	1041	630	579	761.9	796.0
hsa-mir-503	234	331	386	147.8	122.9	U80	476	2972	1204	3594.3	1655.2
hsa-mir-505	1026	1254	1800	560.0	573.2	U81	11963	6655	8135	8048.5	11183.6
hsa-mir-532	1605	2709	3673	1209.8	1169.6	U82	72625	10772	11769	13027.6	16179.5
hsa-mir-542	872	2007	2606	896.3	829.9	U83	4434	849	822	1026.8	1130.0
hsa-mir-548e	787	1702	2241	760.1	713.6	U83A	4218	11391	3298	13776.3	4533.9
hsa-mir-548k	3865	7475	9839	3338.4	3133.1	U83B	1377	511	505	618.0	694.3
hsa-mir-548n	114	288	304	128.6	96.8	U84	6797	1087	1486	1314.6	2042.9
hsa-mir-550-1	1133	2142	3011	956.6	958.8	U85	421	281	308	339.8	423.4
hsa-mir-550-2	1120	2115	2897	944.6	922.5	U86	3308	450	609	544.2	837.2
hsa-mir-551b	251	359	419	160.3	133.4	U87	4467	4350	6323	5260.9	8692.6
hsa-mir-561	2153	4552	6404	2032.9	2039.3	U88	21972	18832	24526	22775.4	33717.2
hsa-mir-573	279	684	882	305.5	280.9	U89	407	677	616	818.8	846.8
hsa-mir-576	692	1170	1281	522.5	407.9	U91	301	64	89	77.4	122.4
hsa-mir-577	14925	28649	42538	12794.7	13545.7	U93	2980	1040	1621	1257.8	2228.5
hsa-mir-578	124	198	249	88.4	79.3	U94	403	172	94	208.0	129.2
hsa-mir-582	1090	2220	3039	991.5	967.7	U95	28698	12440	10165	15044.9	13974.4

hsa-mir-589	5427	12117	14812	5411.5	4716.7	U96a	2552	6188	2510	7483.8	3450.6
hsa-mir-598	439	982	1336	438.6	425.4	U97	5002	1860	1929	2249.5	2651.9
hsa-mir-615	3143	7026	9353	3137.8	2978.4	Z17B	57244	13038	14365	15768.1	19748.3
hsa-mir-616	106	112	160	50.0	51.0						
hsa-mir-625	516	691	668	308.6	212.7						
hsa-mir-627	111	225	237	100.5	75.5						
hsa-mir-628	421	807	957	360.4	304.7						
hsa-mir-629	3251	7999	8677	3572.4	2763.1						
hsa-mir-641	5854	7219	9600	3224.0	3057.0						
hsa-mir-652	1763	4684	6785	2091.9	2160.6						
hsa-mir-660	732	1131	1376	505.1	438.2						
hsa-mir-663	184	31	29	13.8	9.2						
hsa-mir-671	6577	11844	16788	5289.6	5346.0						
hsa-mir-7-1	12388	17492	25285	7812.0	8051.7						
hsa-mir-7-2	26489	40221	52039	17962.8	16571.2						
hsa-mir-7-3	25688	38855	50918	17352.8	16214.3						
hsa-mir-708	2786	4528	6416	2022.2	2043.1						
hsa-mir-744	5545	13564	16077	6057.7	5119.5						
hsa-mir-766	5798	2481	6409	1108.0	2040.9						
hsa-mir-769	27221	48192	68710	21522.7	21879.9						
hsa-mir-874	921	2917	4468	1302.7	1422.8						
hsa-mir-877	370	415	567	185.3	180.6						
hsa-mir-887	885	1249	1978	557.8	629.9						
hsa-mir-9-1	228	415	706	185.3	224.8						
hsa-mir-9-2	293	450	774	201.0	246.5						
hsa-mir-9-3	2688	6899	10142	3081.1	3229.6						
hsa-mir-92a-1	389272	973704	1338081	434859.2	426096.9						
hsa-mir-92a-2	433179	616479	909778	275321.4	289708.6						
hsa-mir-92b	48869	120069	158085	53623.2	50340.4						

hsa-mir-93	43167	65373	95133	29195.8	30294.0
hsa-mir-935	121	322	464	143.8	147.8
hsa-mir-941-1	4072	8600	13609	3840.8	4333.6
hsa-mir-941-2	7553	16236	25904	7251.0	8248.8
hsa-mir-941-3	7558	16071	25787	7177.4	8211.6
hsa-mir-942	1843	3268	4062	1459.5	1293.5
hsa-mir-96	680	2279	3364	1017.8	1071.2
hsa-mir-98	2506	7803	11333	3484.8	3608.9
hsa-mir-99a	5065	16245	22115	7255.1	7042.3
hsa-mir-99b	37834	66732	106837	29802.7	34021.0
