# Supplementary material for: Contribution of natural antisense transcription to an endogenous siRNA signature in human cells
Source: BMC Genomics. 2014 Jan 13;15:19. doi: 10.1186/1471-2164-15-19 (PMC3898206; doi:10.1186/1471-2164-15-19)
Supplement: Additional file 5: Figure S3 — Length distribution of the short RNAs in the three samples control, clone 5 and clone 12. [file 1471-2164-15-19-S5.pdf]

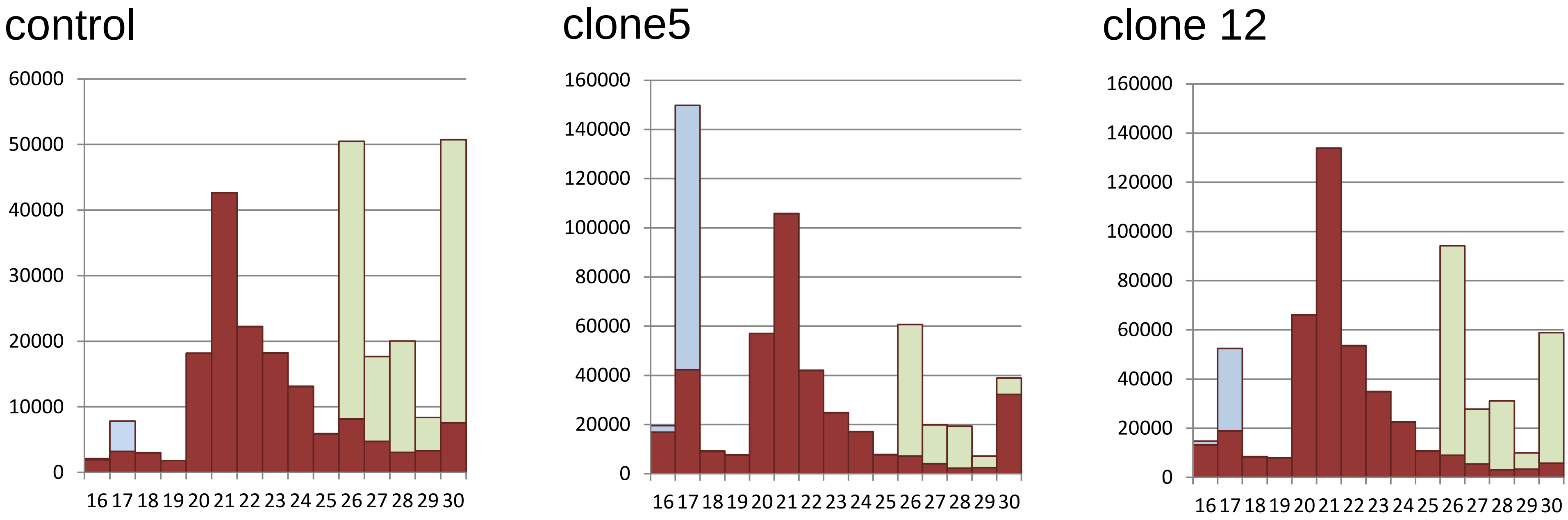

Length distribution of the short RNAs in the three samples control, clone 5 and clone 12. The length of the short RNAs is given on the x-axis, the read counts are on the y-axis. Two genes, STAMBP and TMEM25, contributed disproportionally to the read numbers. STAMBP related reads are represented in blue, TMEM related reads are shown in green. The fact that reads of these two genes are over-represented in all samples points to a cell line inherent bias rather than a mutagen induced change.
