# Supplementary material for: Contribution of natural antisense transcription to an endogenous siRNA signature in human cells
Source: BMC Genomics. 2014 Jan 13;15:19. doi: 10.1186/1471-2164-15-19 (PMC3898206; doi:10.1186/1471-2164-15-19)
Supplement: Additional file 7: Table S4 — Details and sequences of primers and probes for qPCR. [file 1471-2164-15-19-S7.pdf]

**wild type**		orientation						RNAseq reads	
	distance	> + / < -	distance		qPCR			+ strand	- strand
U2AF2>	0.45	**>EPN1>**	**12**	<NLRP9	4.14	5.39	nd	74	90
KATNB1>	1	**<KIFC3<**	**80**		6.91	8.95		39	14
	300	**>KIAA0128>**	**1.45**	<GINS2		3.23	4.57	8	12
ALOXE3<	1.67	**<HES7<**	**16**		11.54	14.98		126	3
	49	**<RABAC1<**	**7.2**	<ATP1A3		5.05	6.9	29	22
RLTPR>	0	**<ACD<**	**0.13**	>PARD6A	8.3	5.61	11.31	3	297
NUP85>	0.84	**<GGA3<**	**0**	<MRPS7	7.14	7.6	5.94	0	24
HLA-DMA<	15	**>BRD2>**	**22**	<HLA-DOA	12.64	2.27	8.52	159	9
NR2F1>	23	**<FAM172A<**	**39**	<KIAA0825	8.09	3.9	9.95	81	3109
	40	**>CCDC86>**	**0**	< GPR44		3.62	nd	0	59

**Clone 5**		orientation						RNAseq reads	
	distance	> + / < -	distance		qPCR			+ strand	- strand
U2AF2>	0.45	**>EPN1>**	**12**	<NLRP9	3.95	5.71	nd	461	8
KATNB1>	1	**<KIFC3<**	**80**		6.01	9.21		183	7
	300	**>KIAA0128>**	**1.45**	<GINS2		2.77	4.78	44	24
ALOXE3<	1.67	**<HES7<**	**16**		12.09	15.46		414	30
	49	**<RABAC1<**	**7.2**	<ATP1A3		5.57	6.05	64	74
RLTPR>	0	**<ACD<**	**0.13**	>PARD6A	7.84	5.12	12.09	36	24
NUP85>	0.84	**<GGA3<**	**0**	<MRPS7	5.97	9.01	5.72	0	30
HLA-DMA<	15	**>BRD2>**	**22**	<HLA-DOA	12.42	2.09	9.32	441	2
NR2F1>	23	**<FAM172A<**	**39**	<KIAA0825	8.29	4.02	8.76	177	4097
	40	**>CCDC86>**	**0**	< GPR44		4.42	nd	3	197

**Clone 12**		orientation						RNAseq reads	
	distance	> + / < -	distance		qPCR			+ strand	- strand
U2AF2>	0.45	**>EPN1>**	**12**	<NLRP9	4.14	5.18	nd	456	2
KATNB1>	1	**<KIFC3<**	**80**		6.79	9.16		249	19

	300	**>KIAA0128>**	**1.45**	<GINS2		2.7	4.81	20	64
ALOXE3<	1.67	**<HES7<**	**16**		12.03	14.75		282	6
	49	**<RABAC1<**	**7.2**	<ATP1A3		5.28	7.34	116	73
RLTPR>	0	**<ACD<**	**0.13**	>PARD6A	8.17	5.52	10.66	24	27
NUP85>	0.84	**<GGA3<**	**0**	<MRPS7	6.51	6.97	5.09	9	39
HLA-DMA<	15	**>BRD2>**	**22**	<HLA-DOA	13.24	1.43	8.48	436	34
NR2F1>	23	**<FAM172A<**	**39**	<KIAA0825	7.62	4.62	10.89	204	5527
	40	**>CCDC86>**	**0**	< GPR44		3.77	nd	1	139
